# The household burden of sleeping sickness: out-of-pocket costs for diagnosis and treatment

**DOI:** 10.1136/bmjgh-2026-025040

**Published:** 2026-10-16

**Authors:** Rian Snijders, Alain Fukinsia, Pathou Nganzobo, Erick Miaka, Epco Hasker, Alain Mpanya, Marina Antillon, Fabrizio Tediosi

**Affiliations:** 1 Institute of Tropical Medicine, Antwerp, Belgium; 2 Swiss Tropical and Public Health Institute, Allschwill, Basel-Landschaft, Switzerland; 3 National Program for the Control of Human African Trypanosomiasis, Kinshasa, Democratic Republic of Congo; 4 Mathematics Institute, University of Warwick, Coventry, UK; 5 University of Basel, Basel, Switzerland

**Keywords:** Human African Trypanosomiasis, Health Services Accessibility, Public Health, Health economics, Control strategies

## Abstract

**Purpose:**

This study estimated out-of-pocket (OOP) expenses associated with Human African Trypanosomiasis (HAT) care in the Democratic Republic of the Congo (DRC) and explored how they influenced care-seeking behaviour and participation in HAT control, aiming to inform effective and financially accessible elimination strategies.

**Methods:**

A sequential mixed-methods study was conducted using 16 SSIs and six focus group discussions, followed by a structured survey of 444 recently tested participants across six health zones. Medical and non-medical expenditures were collected by health structure type and screening strategy (active vs passive). Catastrophic health expenditure (CHE) was defined as OOP costs, excluding food, exceeding 10% or 25% of annual household income.

**Results:**

Payments at health facilities, transport costs and long distances delayed care-seeking, particularly in passive screening (PS). Active screening (AS) was associated with minimal OOP, 93% of visits were cost-free, with a median OOP of US$0.76 among those incurring costs. PS generated higher expenses, only 12% of PS visits were cost-free, with a median OOP of US$9.08 among those with expenditures. Among confirmed cases, median OOP was lower through active (US$9.84) than PS (US$24.23). Nearly 90% of confirmed cases sold assets or borrowed money to cover expenses. CHE was uncommon under average household income (<4%) however 36% of passively detected cases exceeded the 10% threshold under minimum-wage income assumptions.

**Conclusion:**

Despite free diagnosis and treatment, accessing HAT care in rural, low-resource foci in the DRC still imposes a substantial financial burden. Reaching elimination targets and ensuring equitable access will require minimising indirect costs and logistical barriers to screening and diagnosis. As AS declines and routine health systems assume greater surveillance responsibilities, reducing indirect costs and logistical barriers will be critical to sustain coverage and maintain an effective and equitable HAT elimination strategy.

**WHAT IS ALREADY KNOWN ON THIS TOPIC**

- Although gambiense human African trypanosomiasis (gHAT) screening and treatment are provided free of charge in the Democratic Republic of the Congo, patients still incur substantial out-of-pocket costs that can delay timely care-seeking and discourage screening; however, the financial burden of active versus passive screening and the impact on uptake had not been previously compared.

**WHAT THIS STUDY ADDS**

- Passive screening imposes substantially higher costs than active screening, with financial barriers shaping the care-seeking pathway, through reliance on nearby providers unable to diagnose gHAT, delayed care in anticipation of mobile teams and repeated visits before diagnosis; nearly 90% of confirmed cases resorted to selling assets or borrowing to cover expenses, with 36% of passively detected cases exceeding the 10% catastrophic expenditure thresholds under minimum-wage income assumptions.

**HOW THIS STUDY MIGHT AFFECT RESEARCH, PRACTICE OR POLICY**

- As gHAT surveillance increasingly relies on passive detection, elimination strategies should address the impact of patient’s costs to improve coverage by making screening more geographically accessible, community-proximate points of care, particularly in low-income and remote settings, alongside clearer referral pathways, transport support and coverage of other non-medical costs.

## Introduction

 Gambiense human African trypanosomiasis (gHAT) or sleeping sickness is a parasitic disease caused by *Trypanosoma brucei gambiens*e, transmitted by tsetse fly and lethal when untreated.^1^ During the colonial era, it devastated West and Central Africa, decimating villages.^2^ Intensive case management and vector control brought the disease under control, but a resurgence in the 1970s caused nearly 40 000 annual cases by the late 1990s.^3^ Subsequent interventions achieved elimination as a global public health problem (PHP) in 2020, defined by the WHO as fewer than 2000 annual cases continent-wide and under 1 case per 10 000 inhabitants in at least 90% of endemic foci.^4 5^ The current goal is zero human transmission by 2030. In 2024, WHO reported 546 gHAT cases across ten countries, with 60% in the Democratic Republic of the Congo (DRC-S1).^6–8^

gHAT control combines population screening in endemic villages (active screening, AS), testing of self-presenting patients at fixed health facilities (passive screening, PS), and tsetse control in certain regions. Diagnosis begins with serological screening, followed by more complex laboratory tests if positive. Treatment evolved from stage-specific regimens requiring a lumbar puncture for disease staging—pentamidine injections for stage 1 and arsenic-based melarsoprol for stage 2, which caused severe adverse events—to nifurtimox-eflornithine combination therapy for second-stage disease from 2009.^1^ Since 2020, fexinidazole, the first all-oral treatment for both stages has been recommended as first-line therapy except for severe stage disease, eliminating the need for lumbar puncture staging.^2^ In early 2026, the European Medicines Agency approved acoziborole as a single dose treatment for both stages.^9 10^

Although screening and treatment are officially free, patients face indirect and uncovered direct costs, transportation, food and non-HAT medical expenses, limiting access to care.^11–13^ This is important because in 2024, 74.6% of the Congolese population lived below the extreme poverty line (US$2.15 /day),^14^ with two-thirds lacking basic necessities, particularly in rural and conflict-affected areas.^15^ Earlier studies showed that out-of-pocket (OOP) expenditures related to gHAT screening and treatment reduce participation and delay care-seeking. Gouteux and Robays linked such costs to lower AS participation.^11 12^ Lutumba estimated an economic burden of US$164 per case, representing 43% of a rural household’s annual income, with direct medical expenses accounted only for 5%.^13^ These studies reflect the mobile AS and melarsoprol era, while today PS now identifies 40%–50% of cases.^16^ In today’s elimination phase, however, costs associated with screening become increasingly important: if costs discourage screening uptake or delay diagnosis, infections may remain undetected for longer, hampering interruption of transmission and progress towards elimination.

As WHO targets zero gHAT transmission by 2030 under the Neglected Tropical Diseases Roadmap, understanding economic barriers under current screening and treatment strategies becomes increasingly critical.^17 18^ This study assesses the financial gHAT burden on individuals screened through AS and PS, and examines how costs affect care-seeking and participation to inform more effective and equitable gHAT strategies.

## Materials and methods

### Research setting

The study was conducted in Kwilu province, western DRC (78 441 km²) with five territories and 24 health zones. Each health zone serves around 100 000–200 000 people through a reference hospital and peripheral health centres.^19–22^ Kwilu is among DRC’s most economically disadvantaged provinces: over half the population lives in severe poverty, with over 86% lacking reliable access to education, healthcare, clean water or adequate housing.^15^ In 2019, Kwilu reported the country’s highest number of gHAT cases, with several health zones exceeding the PHP elimination threshold of 1 per 10 000 inhabitants (online supplemental information S2).

### Study design

We used an exploratory sequential mixed-methods design, in which qualitative findings informed the quantitative survey’s design and interpretation. Interviews and focus group discussions identified locally relevant cost categories, care-seeking pathways and barriers to screening and treatment. These findings informed survey modules covering medical and non-medical costs, transport, food, income loss, accompanying persons, repeated visits and financial coping strategies.

Integration continued during analysis and interpretation. Qualitative findings guided the classification of health structures into AS and PS pathways and interpretation of costs incurred before or outside specialised gHAT services. The integrated findings described both the magnitude of OOP expenditure and how anticipated and experienced costs influenced care-seeking, delays, screening participation and financial coping.

### Study participants and sample selection strategy

Eligible participants were consenting adults (≥18 years) who participated in gHAT screening. The qualitative phase additionally included healthcare workers and community leaders. All participants provided written informed consent prior to enrolment.

The sampling database combined data from the Programme National de Lutte contre la Trypanosomiase Humaine Africaine (PNLTHA) with health-facility registers. For each village, random lists were generated of adults tested for gHAT during the preceding 6 months, stratified by screening strategy: mobile team visits (AS) or attendance at fixed health facilities (PS).

The survey sample size was determined pragmatically to capture diverse settings and care pathways within available resources and remaining feasible, given a 6-month eligibility window used to limit recall bias.^23^ Three higher- and three lower-endemicity health zones were selected, with two study health areas per zone. No formal power-based sample-size calculation was conducted; the target reflected coverage and feasibility. Purposive recruitment across both screening strategies covered five mutually exclusive screening outcome groups across both strategies: serology negative, serology result unknown, serology performed with microscopy negative, serology positive with microscopy not done or unknown and confirmed gHAT cases defined by parasitological confirmation by microscopy. Sampling sought diversity rather than statistical representativeness, including rare, confirmed cases to capture treatment-related costs. An initial target of 30 participants per area gave 360 participants: recruitment was extended in underrepresented groups to improve the group-specific cost estimates and ensure adequate coverage of relevant cost and care pathways, resulting in 444 eligible participants.

### Qualitative phase

Routine 2020 PNLTHA data were reviewed to identify suitable endemic health zones (online supplemental information S2). Semi-structured thematic guides covered four domains: knowledge and perceptions of gHAT; care-seeking behaviours and therapeutic pathways; the influence of costs on choices; and factors shaping decisions to undergo gHAT screening.^24^

Between May and July 2021, semi-structured interviews (SSIs) and focus group discussions (FGDs) were organised until thematic saturation was reached (online supplemental information S18 and S19). An experienced anthropologist conducted all sessions in Kikongo, the local language, which were audio-recorded, translated and transcribed into French. Transcripts were imported and analysed in NVivo 11 using a codebook based on the topic guides, and iteratively expanded with inductive codes.

### Quantitative phase

The qualitative findings informed a structured survey that was developed in Open Data Kit, combined with standardised paper forms recording all payments (Survey: online supplemental information S20).^25^ In September 2021, six teams of trained local surveyors, accompanied by supervisors, contacted 449 people across six health zones, covering two endemic health areas per health zone (online supplemental information S2).

Health structures were classified by screening strategy. Traditional practitioners, pharmacies, primary care facilities (health posts, health centres or reference centres which are typically staffed by nurses and generally do not have medical doctors on site), private practices, hospitals, Centres for Diagnosis, Control and Treatment (CDTCs) and other fixed structures were grouped as PS, as they rely on self-presenting patients. Most facilities lacked gHAT testing capacity, except for hospitals, CDTCs and some primary care facilities. Truck- or motorcycle-based mobile team visits were classified as AS, reflecting community level screening.

The survey recorded structures visited, visit sequence, symptoms before screening and screening strategy. It did not systematically record the initial reason for seeking care or whether gHAT testing was initiated by the patient or through health-worker referral. For PS, reported costs reflected the overall care-seeking visit by cost category, not gHAT-specific tests or treatment, which are provided free of charge.

All analyses were performed in R (V.2023.06.1).^26 27^ Differences in gHAT symptoms awareness and health facilities visited by screening strategy were assessed using independent two-sample t-tests, with Welch’s correction when variances were unequal.

#### OOP expenses

Costs were recorded in Congolese Francs (CDF) and converted to US$ using the September 2021 exchange rate (US$1=1982 CDF).^28^ Total OOP expenditure per visit was calculated as:

OOP = Medical costs(C_Med_) + Non-medical costs(C_NonMed_)

where

C_Med_ = Consultation + Laboratory tests + Other procedures + Medication + Hospitalisation + Other medical costs

C_NonMed_ = Transport+ Food + Other non-medical costs

For participants reporting multiple visits, expenditures were summed across visits to estimate the total financial burden. At participant level, screening strategy was classified according to the structure that performed the first serological test.

The proportion of zero-cost visits was calculated by screening strategy and compared using chi-square or Fisher’s exact tests. Predictors of any OOP expenditure were examined using binomial logistic regression, with health structure type, travel time, transport mode and screening outcome as predictors. Among participants reporting non-zero expenditure, a generalised linear model with a gamma distribution and log link assessed factors associated with expenditure level. Model fit was evaluated using likelihood ratio tests.

Logistic regression diagnostics included residual plots, influential observations and multicollinearity were evaluated. Gamma model diagnostics included deviance residuals, residual-versus-fitted plots, normal Q–Q plots of deviance residuals and influential observations.

OOP results are presented as adjusted ORs with 95% CIs. Given right-skewed expenditures, costs among visits and participants with non-zero expenditures were summarised using medians, plotted on a logarithmic scale, and compared using Kruskal–Wallis tests.

#### Catastrophic health expenditure

Catastrophic health expenditure (CHE) occurs when OOP payments exceed a household’s capacity to pay. Because reliable household consumption or income data for the DRC were unavailable, the 2021 Anker Living Income Reference Value for rural DRC was used as proxy. The value used was contemporaneous with data collection. This reference estimates average monthly household consumption at 310 181 CDF (US$158 monthly; US$1896 annually), compared with 54 224 CDF (US$28 monthly; US$336 annually) for households relying on minimum-wage earnings, assuming a six members reference family with 1.77 full-time-equivalent workers.^29^ CHE was defined using the budget-share approach: OOP expenditure (excluding food) exceeding 10% or 25% of estimated annual household income.^30^

### Patient and public involvement

Community members and gHAT-affected individuals participated as research subjects but were not partners in developing the research questions or analytical approach. Findings will be disseminated to the national gHAT control programme and relevant health authorities to inform decisions aimed at reducing financial barriers to gHAT care.

### Reporting guideline compliance

The quantitative cross-sectional component was reported in accordance with Strengthening the Reporting of Observational Studies in Epidemiology (STROBE). Reporting of the sequential mixed-methods design was additionally guided by the six Good Reporting of A Mixed Methods Study (GRAMMS) criteria and the compliance table provided in online supplemental information S21.

## Results

Results are presented sequentially: qualitative findings first describe care-seeking pathways and perceived financial barriers, followed by quantitative estimates of the frequency, magnitude and consequences of OOP expenditure.

### Qualitative phase

Data collection continued until thematic saturation, yielding 6 FGDs and 16 SSIs (details: online supplemental information S3). The analysis summarised key themes used for the development of the quantitative survey.

#### Pathways to care: gHAT awareness and symptom response

Community members demonstrated varying levels of gHAT awareness. Symptoms like somnolence, weakness and body pain were commonly mentioned, although knowledge of transmission and specific symptoms remained limited. gHAT was often described as life-threatening, illustrated by accounts of death among undiagnosed or untreated community members.

Participants reported first using pharmacy medication when unwell and visiting health facilities only after three or more days if symptoms worsened. They recommended seeking care sooner for young children, perceived as more vulnerable. Over-the-counter medications like paracetamol were used without a diagnosis. Health centres or hospitals were preferred for persistent or worsening symptoms, though several barriers limited their accessibility.

#### gHAT screening, diagnosis and treatment

Most participants knew that screening was available through mobile teams visiting villages (AS) or fixed health facilities (PS). Many participants had experienced AS, either door-to-door by motorbike teams or village-centred by truck-based teams. Some preferred door-to-door visits for greater privacy, shorter waiting times and better fit with daily routines.

For PS, participants reported that local health centres often lacked appropriate equipment (microscope) to diagnose gHAT. Fixed diagnostic services were perceived as scarce and located in distant hospitals.

#### Economic burden and implications of gHAT screening and treatment

All participants said that AS was free, some recalled previous card fee. They also mentioned that after AS campaigns, village chiefs sometimes encouraged voluntary food contributions as thanks.

Regarding PS, respondents reported that while gHAT tests were free, health workers would refuse care until initial payments, registration or patient file fees were made, leading to delays or avoidance, even when formal medical attention was recognised as necessary.

Money is a barrier to care because when you arrive at the health centre, before they even touch you, you first have to buy a form, then come the tests. If you don't have the money, you don't go, which is why my mom keeps us at home even when we feel very sick (FGD with women, gHAT endemic village)

Hospital visits were considered more expensive, and less accessible, than primary care, requiring extensive preparation, including travel, childcare and time off work. Participants described selling livestock or belongings to afford care.

I can give them(health care staff) a goat. …, among the nurses, whoever has money can pay for you and take the goat or the chicken, depending on what you have. …., my wife was sick, but I didn't have any money, so we brought a piece of cloth we had at home so that they could treat my wife … (SSI, adult male, gHAT endemic village)

Fear of unaffordable care or refusal of services led participants to delay or defer care: expected payments ranged from 2500 CDF (≈US$1.26) to nearly 200 000 CDF (≈US$100). Participants also reported non-medical costs including motorcycle travel, fuel, food, lodging, registration, supplementary diagnostics and lost income.

I have five children. If one of them has sleeping sickness and I have to go to the Mission for treatment, …, I have to take the other four children with me. Otherwise, who would feed them? If I left them in the village, the worst could happen. (FGD with women, gHAT endemic village)

#### Barriers to gHAT screening

Non-participation in AS was attributed less to unwillingness than to fear, stigma and practical barriers. Fear of social exclusion, income loss or extended absence from home outweighed perceived benefits of early diagnosis. Household and fieldwork responsibilities took priority over care. Fear of painful procedures, like lumbar puncture, public identification as sick, dietary restrictions, mistrust and misinformation, including confusion between gHAT and HIV screening, also discouraged participation. One participant highlighted the *chef du village*, the traditional village leader‘s influential role in mobilising community participation. Limited engagement or trust by this authority could hinder uptake.

Delays between screening and diagnosis were common, particularly when travel to distant confirmation centres was required. Although treatment was free once diagnosed, poor roads, unavailable transport and long distances hampered access, sometimes requiring up to 2 days of walking. Some suspected cases waited for mobile teams rather than seeking care in facilities, further delaying diagnosis and treatment.

… the child doesn't know how to come here for treatment because the Mission is very far away; he can't walk and has no means of transportation. (SSI, adult male former gHAT patient, gHAT endemic village)

Clinical trial sites were perceived as offering higher-quality care, better infrastructure and free non-gHAT-related medical care, food and other services, leading some patients to delay treatment or travel farther to access them.

now they send a car or motorcycle to take you to where they treat sleeping sickness. They will give you food, and everything is completely free, even your return to the village after treatment. (FGD with men, gHAT endemic village)

Despite these challenges, most participants were willing to use and recommend gHAT services, particularly when they were accessible, affordable and clearly explained.

### Quantitative phase

#### Sample description

Of 449 individuals contacted, 444 (99%) were eligible and included in the analysis (table 1). Five were excluded due to absence, lack of consent or inability to recall gHAT screening. The population included 57% women, with nearly 80% under 50 years old. Around half were screened through each strategy. Confirmed and seropositive participants were purposively oversampled relative to their prevalence, which rarely exceeds 1–2% in high-endemic settings, to represent the full cost spectrum. Overall, 10% were confirmed gHAT cases, 21% had positive serology with negative microscopy, 59% were seronegative and approximately 10% had incomplete or unknown outcomes.

**Table 1:** Description of study population

	Active (n=230)	Passive (n=214)	Excluded from analysis (n=5)	Overall (n=449)
Population contacted				
Study population				
Absent	0 (0%)	0 (0%)	2 (40%)	2 (0.4%)
No informed consent	0 (0%)	0 (0%)	1 (20%)	1 (0.2%)
No recall gHAT screening	0 (0%)	0 (0%)	2 (40%)	2 (0.4%)
Eligible for inclusion	230 (100%)	214 (100%)	5 (100%)	444 (98.9%)
Population included				
Sex				
Male	102 (44.3%)	86 (40.2%)		188 (42.3%)
Female	128 (55.7%)	128 (59.8%)		256 (57.7%)
Missing	0 (0%)	0 (0%)		0 (0%)
Age category				
Young adults: 18–30 years	106 (46.1%)	91 (42.5%)		197 (44.4%)
Middle-aged adults: 31–50 years	71 (30.9%)	82 (38.3%)		153 (34.5%)
Older adults: 51–70 years	47 (20.4%)	36 (16.8%)		83 (18.7%)
Seniors: 71+years	6 (2.6%)	5 (2.3%)		11 (2.5%)
Missing	0 (0%)	0 (0%)		0 (0%)
Screening outcome				
Confirmed gHAT case	18 (7.8%)	28 (13.1%)		46 (10.4%)
Serology: result unknown	10 (4.3%)	11 (5.1%)		21 (4.7%)
Serology performed; microscopy negative	61 (26.5%)	32 (15.0%)		93 (20.9%)
Serology: negative	128 (55.7%)	134 (62.6%)		262 (59.0%)
Serology: positive; microscopy: not done/unknown	13 (5.7%)	9 (4.2%)		22 (5.0%)

gHAT, gambiense human African trypanosomiasis.

#### Knowledge of gHAT and health-seeking behaviour

Participants identified on average three gHAT characteristics (online supplemental information S4), most commonly headaches, sleep disorders and fever, with no significant difference between AS and PS (t=−0.92, df=442, p=0.356). Awareness of community gHAT cases was higher among AS (88%) than PS participants (73%)(online supplemental information S5).

Overall, 60% of participants reported perceived gHAT symptoms before screening, more commonly in PS (83%) than AS (38%) (online supplemental information S6). Among symptomatic participants, 65% sought care before screening (AS: 55/88; PS: 119/178), first attending a primary care facility (45%) or pharmacy (41%). Hospitals were rarely a first choice (9%), with over 80% using specialised services (mobile teams or CDTC) only after exhausting other options (online supplemental information S7). In PS, gHAT testing may also have been initiated by health workers based on clinical judgement or local epidemiological risk, including when participants did not perceive their symptoms as gHAT-related.

The 444 participants reported 800 visits (online supplemental information S8–S10). Mean visits were slightly higher in AS (1.92) compared with PS (1.67; t=2.34, df=422.9, p=0.02). Pharmacies and mobile teams were most accessible, with over 95% of visits requiring less than 1 hour on foot. Primary care facilities were also generally accessible (71% within 1 hour) by motorcycle (47%), walking (30%) or biking (20%). Almost half of hospital visits required half to a full day of travel, primarily by motorcycle (49%) or on foot (31%).

Income loss was reported by 54% during screening (241/444), 36% during microscopy confirmation (53/146) and 89% during treatment (41/46), and frequent in PS (online supplemental information S11). Agriculture and crop sales were the main source of income, followed by fishing, around 30% combined farming, fishing, small-scale livestock or selling palm products. Only 9 out of 444 had health insurance, and the sole insured gHAT case still incurred costs.

#### OOP costs

PS was associated with a greater likelihood and magnitude of OOP expenditure than AS (figure 1 and online supplemental information S12). Among AS visits, 93% were cost-free. Paying AS visits had a median OOP of US$0.76, including food (online supplemental information 12), US$0.62 for vehicle-based and US$2.02 for motorcycle-based AS. By contrast, 88% of PS visits generated costs, with a median OOP for paying visits of US$9.08 (online supplemental information 12), ranging from US$2.52 at pharmacies to US$33.68 at hospitals.

**Figure 1:**
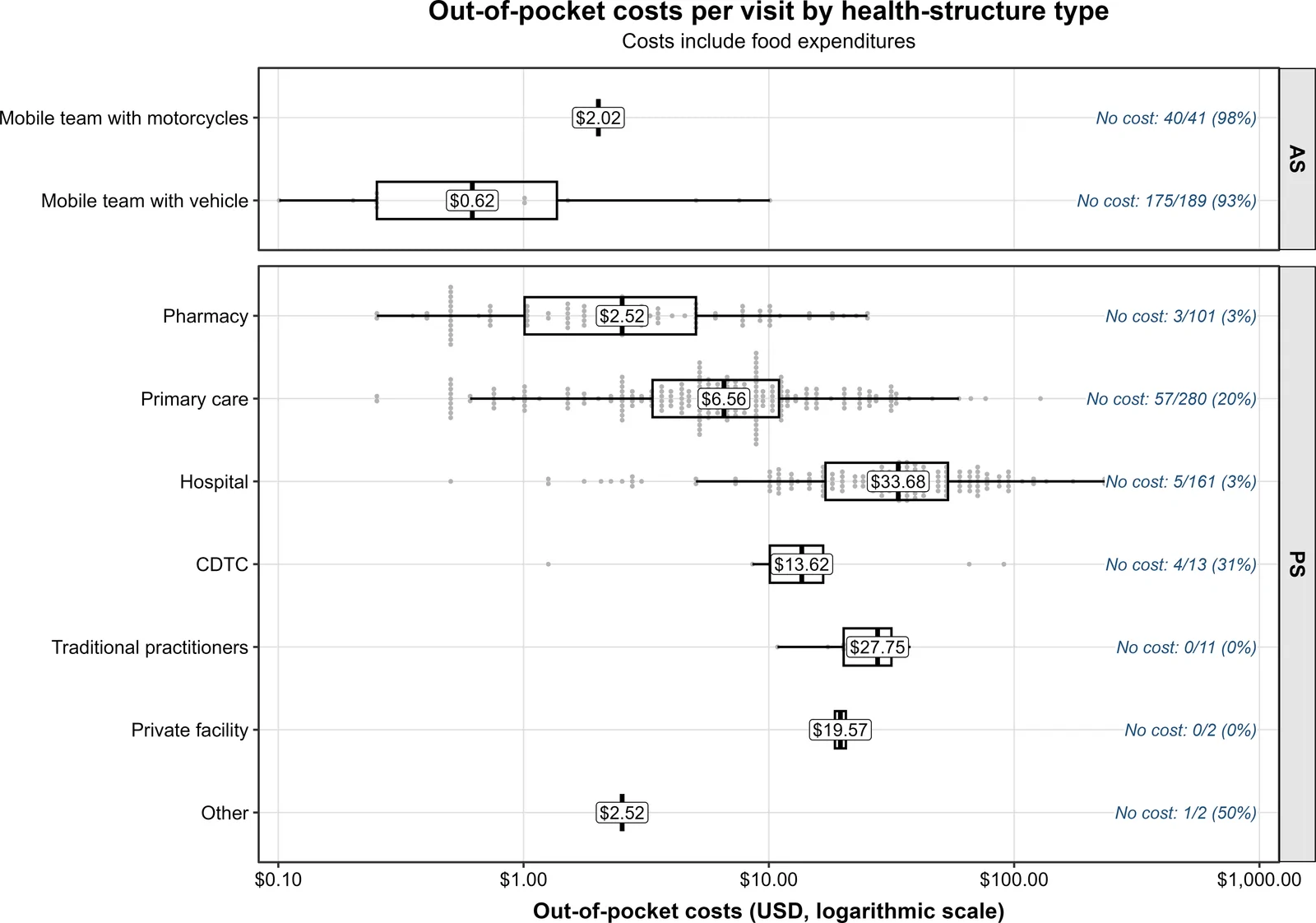
Boxplot of the median out-of-pocket expenses per visit by health structure type (visits with OOP>0, including food costs) (n=800). Primary care: health post or health centre, generally staffed by nurses. Hospital: secondary or primary referral hospital. 285/800 visits involved no out-of-pocket costs. AS, active screening; CDTC, HAT screening, diagnosis and treatment centre; HAT, human African trypanosomiasis; OOP, out-of-pocket; PS, passive screening.

Health structure type was the strongest predictor of incurring OOP: visits to pharmacies, hospitals and primary care facilities showed substantially higher odds (OR 42–442) than to AS visits (online supplemental information 12). In the full binomial model, health-structure type was strongly associated with the probability of incurring OOP, while travel time was borderline (p=0.057); transport mode and screening outcome were not significant. Among paying visits, the regression model showed associations of health-structure type and screening outcome with higher expenditure levels, but not travel time nor transport mode (online supplemental information S13 and S14). Across strategies, medical care accounted for approximately one-third of total OOP, and food and transport for another third (online supplemental information S15 and S16).

Figure 2 shows participant-level OOP by screening outcome and strategy for all 444 participants (online supplemental information 12). Across outcome groups, 22%–77% of AS participants incurred no costs, compared with 0%–34% in PS. Among confirmed cases, median OOP was more than double in PS (US$24.23) than in AS (US$9.84); every PS-case incurred costs, compared with 78% in AS. Among participants reporting costs, seronegative participants had median OOP of US$20.18 in PS, and US$18.52 but far fewer PS participants were cost free (AS 51% cost-free vs 11% in PS). These costs represent cumulative care-pathway costs, not the serological testing itself, which is provided free of charge, including prior or additional visits to pharmacies, primary care facilities, hospitals or other fixed structures, and transport, food and other expenses. Thus, seronegative, or microscopy-negative participants could report substantial costs, incurred before gHAT screening or outside the mobile campaign. In AS, 86% requiring follow-up microscopy tests received it during the same mobile visit, limiting additional travel (online supplemental information S9).

**Figure 2:**
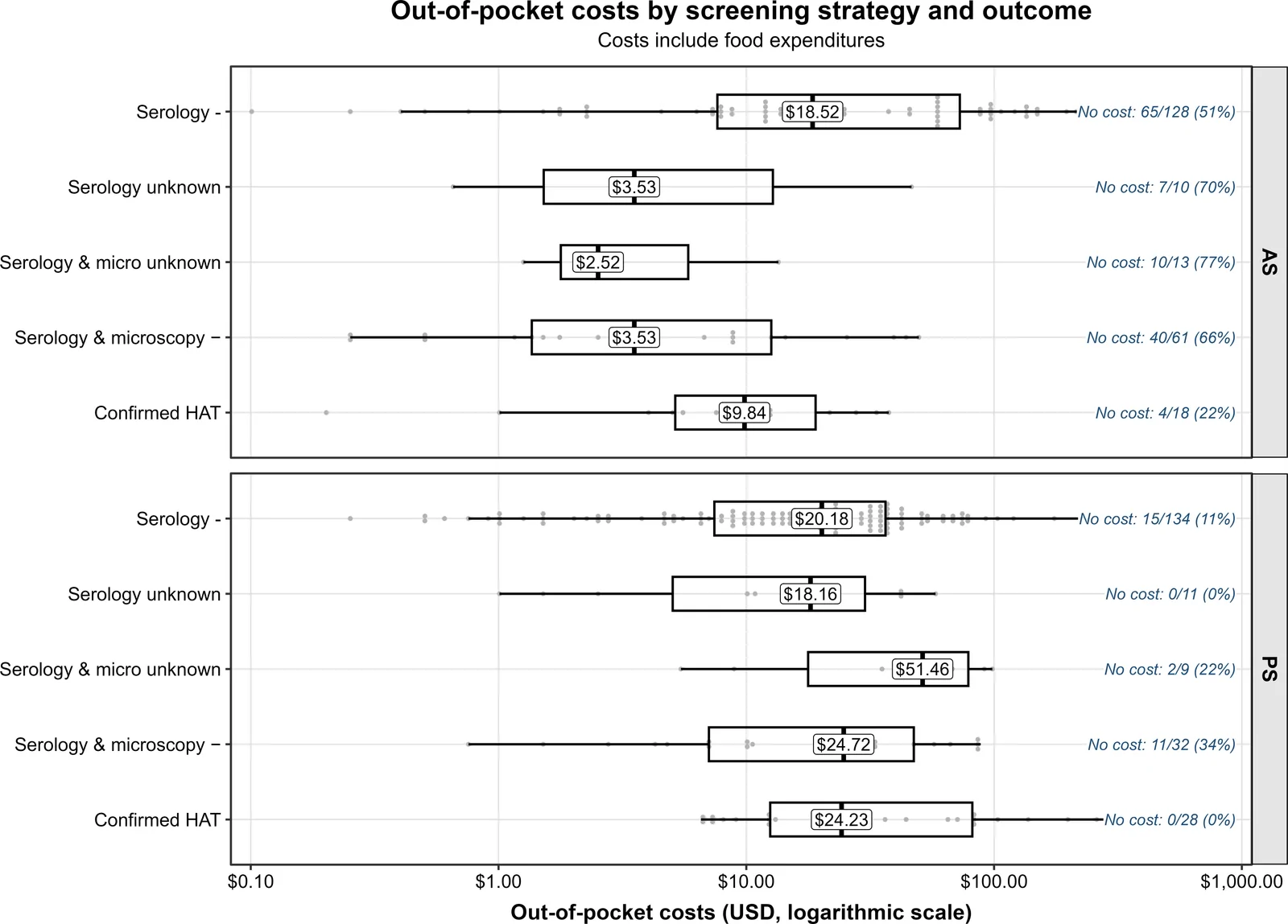
Boxplot of the median out-of-pocket expenses by screening outcome by screening strategy (visits with OOP>0—including food costs) (n=444). Boxplots and medians are based on visits with costs greater than zero; no-cost labels show the number and 154/444 visits involved no out-of-pocket costs. AS, active screening; HAT, human African trypanosomiasis; PS, passive screening.

#### Confirmed cases

The 46 confirmed cases reported a median of two health structure visits before diagnosis and treatment (online supplemental information S17). Nearly all (87%) reported symptoms before screening (AS: 14/18, 78%; PS: 26/28, 93%) and 44 of 46 required hospitalisation. While 40% began treatment immediately, 24% waited one to 2 weeks after reaching the treatment facility. Most (76%) were hospitalised ten days or more, and 43 of 46 were accompanied by a family member.

Only four cases, diagnosed through AS, reported no OOP expenditure. AS-detected cases had a median OOP of US$9.84 versus US$24.53 for PS-detected cases (online supplemental information 12). Median OOP rose with diagnostic delay: US$8 for diagnosis at the first visit, US$21 at the second, US$44 at the third and US$141 at the fourth.

Among confirmed cases, medical costs accounted for 57% of average OOP, driven primarily by non-gHAT medication (22%) and consultation fees (16%), food (32%) and transport (10%), comprised the remainder (figure 3). Almost three-quarters sold assets, either alone (48%) or with other strategies (24%), with smaller proportions relying on family contributions (9%) or borrowing (6%).

**Figure 3:**
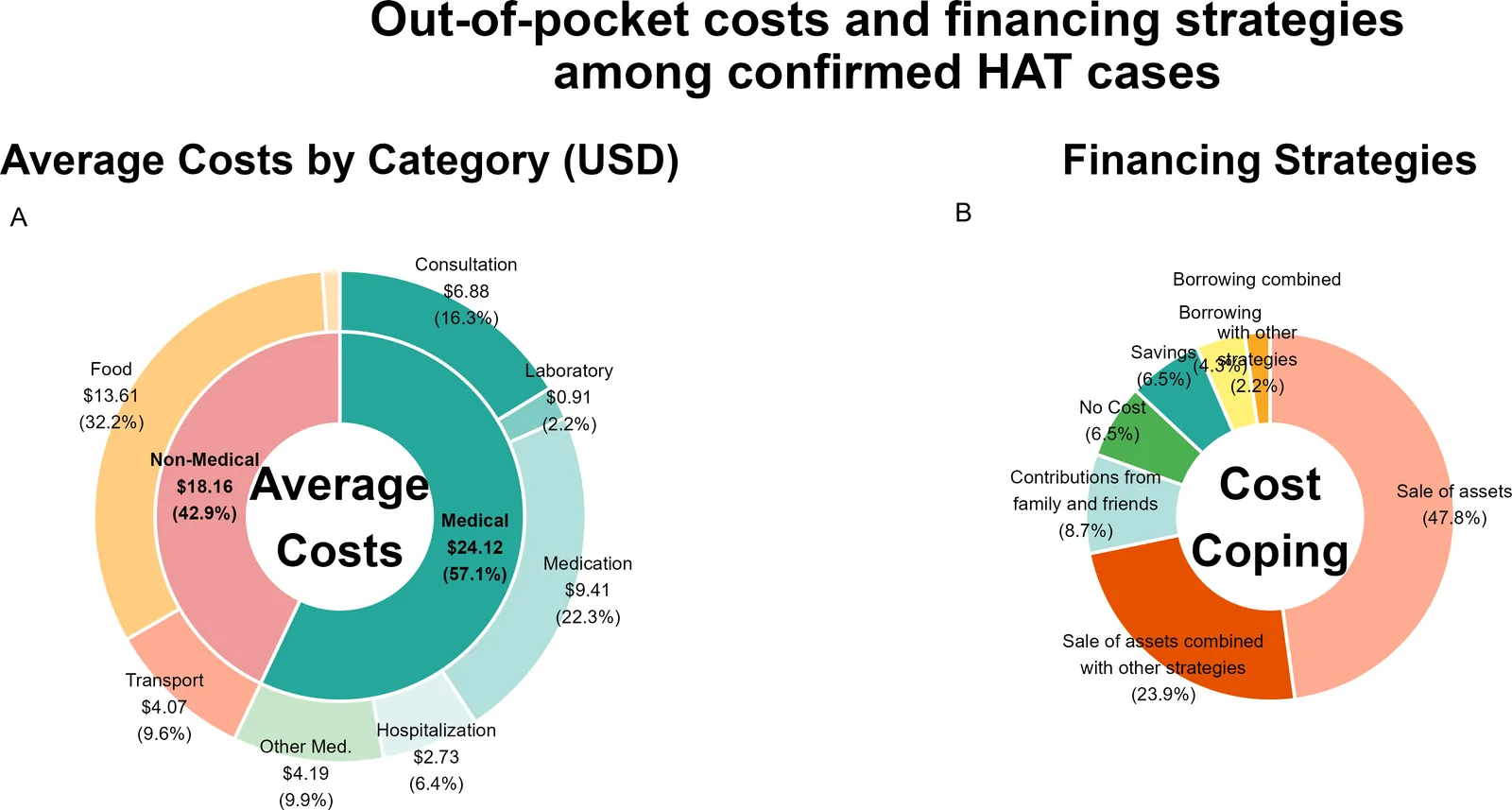
Out-of-pocket expenses (US$) and financial coping strategies for confirmed gHAT cases. (**A**) Average cost chart: Inner ring=sum of component averages; (**B**) Coping strategies for OOP diagnostic and treatment costs. gHAT, gambiense human African trypanosomiasis; HAT, human African trypanosomiasis; OOP, out-of-pocket.

#### Catastrophic health expenditure

Based on the Anker Living Income Reference Value for rural DRC, annual CHE thresholds were US$190–474 for an average rural household and US$34–85 for a minimum wage household. Table 2 shows median OOP excluding food for all cost and no-cost visits, by screening strategy and outcome, and CHE prevalence at 10% and 25% of minimum and average income.

**Table 2:** CHE prevalence by screening strategy and diagnostic outcome across income levels

Outcome	N	No OOP (%)	Median (IQR, US$)	MW 10%	MW 25%	AW 10%	AW 25%
Active screening							
Serology: negative	128	53	0 (0, 12.11)	19	9	1	0
Serology: result unknown	10	70	0 (0, 0.49)	0	0	0	0
Serology: positive; microscopy: not done/unknown	13	77	0 (0, 0)	0	0	0	0
Serology performed; microscopy negative	61	70	0 (0, 1.26)	2	0	0	0
Confirmed gHAT case	18	39	2.02 (0, 5.42)	0	0	0	0
Passive							
Serology: negative	134	16	12.24 (2.59, 26.24)	15	3	1	0
Serology: result unknown	11	0	12.87 (3.78, 24.47)	18	0	0	0
Serology: positive; microscopy: not done/unknown	9	22	8.93 (5.05, 36.33)	44	0	0	0
Serology performed; microscopy negative	32	34	4.54 (0, 18.29)	16	0	0	0
Confirmed gHAT case	28	7	19.30 (7.06, 66.13)	36	14	4	0

Thresholds: MW 10%=US$34; MW 25%=US$85; AW 10%=US$190; AW 25%=US$474.

AW, average wage household (US$1900/year); CHE, catastrophic health expenditure, defined as OOP exceeding specified percentage of annual household income; gHAT, gambiense human African trypanosomiasis; MW, minimum wage household (US$340/year); OOP, out-of-pocket.

AS participants carried the lowest burden, with the overall median OOP at US$0.76, given that 39%–77% incurred no costs at all, and fewer than 1% exceeded CHE thresholds for average-income households. Among seronegative AS participants, 19% exceeded the 10% minimum-wage threshold, probably because of costs at fixed facilities visited outside AS.

PS participants incurred higher OOP with only 7%–34% avoiding OOP, confirmed cases had a median OOP of US$19.30 (IQR: 7.06–66.13]). PS expenditures rarely exceeded CHE thresholds for average-income households (<4%), but approximately one-fifth of PS participants exceeded the 10% minimum income threshold, rising to 36% among confirmed cases. At the stricter 25% threshold for minimum-income households, 14% of confirmed PS cases experienced CHE.

## Discussion

This study provides contemporary, strategy-specific estimates of OOP expenditure for gHAT screening, diagnosis and treatment in rural DRC, extending previous research by comparing AS and PS for the first time.^11–13^ Integrating qualitative data with quantitative findings showed that OOP expenditure is both a financial burden and a driver of delayed care-seeking, selective screening participation, and reliance on cheaper or more accessible providers before reaching specialised gHAT services.

Although gHAT screening is officially free, participants incurred substantial expenses. In AS, median OOP was low with 93% of visits cost-free. In PS, median expenditure was US$20.51 overall and US$24.52 among confirmed cases. PS expenditures by visit and health-structure type generally exceeded estimates for routine general outpatient care in the DRC, approximately US$6.70 nationally and US$13.60 in Bandundu in constant 2010 US$, equivalent to approximately US$16.90 in 2021 US$ after adjustment for inflation.^31 32^ Although OOP rarely exceeded CHE thresholds for average rural households, 36% of PS confirmed gHAT patients exceeded the 10% threshold based on minimum-wage income.

Overall, 89% of gHAT patients borrowed, sold assets or sought financial help. Only 9 of 444 participants had health insurance, and the only insured gHAT patient still incurred expenses. This illustrates the limited inclusion of gHAT endemic populations in Universal Health Coverage efforts, consistent with reports for DRC population that estimate that community-based insurance schemes exist but cover only a small fraction of people, while private insurance mainly serves formal-sector employees and wealthier urban populations.^31 33 34^ Limited financial protection likely leads to delayed or foregone care, worse health outcomes and financial hardship.

These findings are important as gHAT elimination strategies will increasingly depend on PS as AS scales back. Awareness of gHAT is slightly higher among AS than PS participants (88% vs 73%). As cases become rarer, first-hand knowledge will fade, reducing risk perception and community motivation to participate in screening, particularly in population-wide campaigns. Greater reliance on PS may partly shift responsibility and costs to symptomatic individuals seeking care at fixed facilities. Although gHAT services remain free, transport, consultations, medicines and repeated visits can generate substantial OOP costs. Surveillance to achieve a public-health goal should not disproportionately burden rural and low-income populations. Elimination strategies should therefore minimise patient costs, for example, through decentralised diagnostic capacity, clear referral pathways, transport support and communication on expected costs.

The limitations of PS in gHAT endemic, rural, low-income settings shift part of the programmatic burden onto patients. Symptomatic individuals often first seek care at low-cost, nearby options like pharmacies (41%) and primary care facilities (45%), which generally cannot diagnose gHAT beyond assessing symptoms. Hospitals capable of diagnosis were rarely the first point of contact (9%), due to distance and cost, over 45% of considered a hospital only after exhausting other options. Some symptomatic patients postponed care-seeking, waiting for mobile teams despite recognising the risks. Specialised gHAT treatment centres were mentioned as a last resort because patients did not recognise them as services distinct from the hospitals in which they were located. These findings suggest that reliance on PS may delay diagnosis when services are concentrated in hospitals or specialised centres. This is consistent with evidence from the DRC showing that delayed or foregone care is commonly driven by financial constraints, self-medication and distance to facilities. Financial constraints predominated in Equateur, while both financial barriers and distance were important drivers in Maniema/Orientale.^31^

Geographic accessibility further constrains PS uptake. Over 75% of gHAT patients reached facilities within an hour on foot (≈5 km), suggesting that PS primarily serves populations within a 5–10 km radius of diagnostic facilities, excluding at-risk individuals beyond this range. Simarro *et al* estimated that 41% of Africa’s gHAT at-risk population lives within 1 hour of a screening facility and 71% within 3 hours.^35^ Although WHO reported more PS sites in the DRC in 2024, accessibility models typically assume vehicle or motorcycle travel along roads and tracks, overlooking transport affordability and availability in remote, low-resource settings.^5^

Several limitations should be noted. First, income data were not collected due to technical complexity and social sensitivity. Instead, the Anker Living Reference Value was used. Second, lost income, productivity or caregiving costs were excluded, likely underestimating the true economic burden. Third, for PS, the survey captured overall care-seeking costs by category but not the initial reason for care seeking or how gHAT testing was initiated. The expenditures therefore should not be interpreted as costs of gHAT-specific tests or treatment medicines, which are free. Fourth, the low reported use of traditional practitioners reflects the inclusion criteria: participants were only eligible after accessing formal gHAT screening structures. People who consulted traditional practitioners or private facilities without subsequently being referred to or attending a screening facility were not captured. Finally, exchange rate fluctuations (US$1=2896 CDF; +46% in 2024 vs July 2021) may have affected recorded OOP values, as internationally traded goods (drugs, fuel for transport) rose faster than local wages, therefore increasing the real burden.

We recommend that actions prioritise financial accessibility and patient awareness. OOP expenses, including transport, food and uncovered medical fees, should be explicitly discussed with stakeholders and the community to communicate the actual costs patients face. For continued case detection and disease surveillance mobile screening remains important in rural, low-income foci. Although AS poses a limited financial burden and is generally affordable even for the poorest households, fear of hidden costs may still discourage participation. Clear, standardised communication on the financial implications of screening and treatment, as well as available financial or logistical support, could reduce misconceptions and encourage earlier care-seeking.

However, maintaining stand-alone AS may become unsustainable as gHAT approaches elimination, while declining perceived risk of the disease will reduce community participation as other health and livelihood priorities take precedence. Integrating other medical services into gHAT screening, offering community-level incentives or village-level support could improve affordability and uptake. Ultimately, integrating gHAT activities within broader health programmes and surveillance systems will be essential to optimise resources and ensure long-term sustainability.

New treatments might offer solutions. Fexinidazole, a 10-day oral treatment for both disease stages, still requires parasite confirmation and WHO guidelines recommend supervised intake, meaning clinic services remain necessary.^36^ Acoziborole, a single-dose oral treatment for both stages, received a positive scientific opinion from the EMA in February 2026. Acoziborole could be a game changer by reducing costs and burden on patients and the healthcare system by enabling point-of-care treatment and reducing hospitalisation.^10^ If ongoing ‘screen and treat’ trials confirm its safety and feasibility, treatment could rely on serological testing alone, eliminating microscopy tests.^10 37–39^

To strengthen access, early diagnosis and surveillance, opportunities to decentralise screening should be investigated. For instance, dried blood sample collection could be integrated in primary care facilities and pharmacies where patients first seek care. Samples could be periodically picked up for centralised laboratory testing, with coordination and positive case follow-up by the national control programme.^40^ This approach could leverage existing care-seeking patterns rather than requiring patients to travel to distant facilities.

## Conclusion

Achieving WHO’s 2030 goal of zero gHAT transmission will require more than free medication and diagnostics. Over 15% of rural minimum-wage households still face CHE in PS and elimination strategies increasingly dependent on passive surveillance face fundamental challenges in rural, low-income settings. Increasingly, populations will encounter fewer cases, reducing disease awareness. Moreover, symptomatic individuals seek care first at nearby, low-cost facilities unable to diagnose gHAT, and few will travel more than 1 hour to more costly hospitals capable of diagnosis. Ensuring financial protection for the poorest and most remote populations will therefore be critical to sustain engagement in screening and treatment. Strengthening affordability, decentralising diagnosis to facilities where patients already seek care and integrating gHAT activities within broader health services will enhance access and efficiency. Continued investment in community awareness, transport support and patient-centred approaches will be key to maintaining participation as disease prevalence declines. Ultimately, equitable and financially accessible care is essential for achieving and sustaining gHAT elimination.

## Supplementary material

10.1136/bmjgh-2026-025040 online supplemental file 1

10.1136/bmjgh-2026-025040 online supplemental file 2

## Data Availability

Data are available upon reasonable request.

## References

[1] (2013). Control and surveillance of human African trypanosomiasis, WHO technical report series no 984.

[2] Headrick DR (2014). Sleeping sickness epidemics and colonial responses in East and Central Africa, 1900-1940. PLoS Negl Trop Dis.

[3] Cox FEG (2004). History of sleeping sickness (African trypanosomiasis). Infect Dis Clin North Am.

[4] (2023). Factsheet: trypanosomiasis, human African (sleeping sickness). https://www.who.int/news-room/fact-sheets/detail/trypanosomiasis-human-african-(sleeping-sickness).

[5] Franco JR, Priotto G, Paone M (2024). The elimination of human African trypanosomiasis: Monitoring progress towards the 2021–2030 WHO road map targets. PLoS Negl Trop Dis.

[6] (2025). Human African trypanosomiasis: number of new reported cases of human African trypanosomiasis (T.b. gambiense), 2024. https://apps.who.int/neglected_diseases/ntddata/hat/hat.html.

[7] (2025). Human African trypanosomiasis: number of new reported cases of human African trypanosomiasis (T.b. rhodesiense), 2024. https://www.who.int/data/gho/data/indicators/indicator-details/GHO/number-of-new-reported-cases-of-human-african-trypanosomiasis-(t-b-rhodesiense).

[8] Franco JR, Cecchi G, Paone M (2022). The elimination of human African trypanosomiasis: Achievements in relation to WHO road map targets for 2020. PLoS Negl Trop Dis.

[9] (2026). First new chemical entity developed by DNDi and the first all-oral cure for T.b. gambiense sleeping sickness. https://dndi.org/research-development/portfolio/fexinidazole/.

[10] European Medicines Agency (2026). New single-dose oral treatment for human African trypanosomiasis (sleeping sickness). https://www.ema.europa.eu/en/news/new-single-dose-oral-treatment-human-african-trypanosomiasis-sleeping-sickness.

[11] Gouteux JP, Bansimba P, Noireau F (1987). Coût du traitement individuel de la trypanosomiase à Trypanosoma brucei gambiense dans un foyer du Niari (Congo). Med Trop (Mars).

[12] Robays J, Lefèvre P, Lutumba P (2007). Drug toxicity and cost as barriers to community participation in HAT control in the Democratic Republic of Congo. Trop Med Int Health.

[13] Lutumba P, Makieya E, Shaw A (2007). Human African trypanosomiasis in a rural community, Democratic Republic of Congo. *Emerg Infect Dis*.

[14] World Bank Group (2024). The World Bank in the Democratic Republic of Congo. https://www.worldbank.org/en/country/drc/overview.

[15] (2023). Global MPI country briefing 2023: Congo, Democratic Republic of the (Sub-Saharan Africa).

[16] Programme national de lutte contre la trypanosomiase humaine africaine en DRC (PNLTHA) (2025). Rapports annuels: 2014-2024.

[17] Forbes K, Basáñez M-G, Hollingsworth TD (2023). Introduction to the special issue: challenges and opportunities in the fight against neglected tropical diseases: a decade from the London Declaration on NTDs. *Philos Trans R Soc Lond B Biol Sci*.

[18] Lenk EJ, Redekop WK, Luyendijk M (2018). Socioeconomic benefit to individuals of achieving 2020 targets for four neglected tropical diseases controlled/eliminated by innovative and intensified disease management: Human African trypanosomiasis, leprosy, visceral leishmaniasis, Chagas disease. PLoS Negl Trop Dis.

[19] Ministère de la Santé Publique de la République Démocratique du Congo (2012). Arrêté ministériel no 1250/CAB/MIN/SP/CJ/OMK/2012 du 3 Novembre 2012 portant réorganisation des divisions provinciales de la santé en République Démocratique du Congo.

[20] Bruneau J-C (2009). Les nouvelles provinces de la République Démocratique du Congo: construction territoriale et ethnicités. Espacepolitique.

[21] Cellule d’Analyses des Indicateurs de Développement (2017). Province du kwilu: présentation de la province. https://www.caid.cd/index.php/donnees-par-province-administrative/province-de-kwilu/?donnees=fiche.

[22] Ministère de la Santé Publique (2006). Recueil des normes de la zone de santé.

[23] Lakens D (2022). Sample Size Justification. Collabra.

[24] Bukachi SA, Mumbo AA, Alak ACD (2018). Knowledge, attitudes and practices about human African trypanosomiasis and their implications in designing intervention strategies for Yei county, South Sudan. PLoS Negl Trop Dis.

[25] (2015). Protocol for survey to determine direct and indirect costs due to TB and to estimate the proportion of TB-affected households experiencing catastrophic total costs due to TB.

[26] Posit Team (2023). RStudio: integrated development environment for R. Version 2023.06.1.

[27] R Core Team (2024). R: a language and environment for statistical computing. Version 4.4.0.

[28] Free Currency Rates (2021). United States dollar and congolese franc exchange-rate history for 2021. https://freecurrencyrates.com/en/exchange-rate-history/USD-CDF/2021.

[29] N’Guessan YG, Anker R, Anker M (2021). Anker living income reference value for rural Democratic Republic of Congo.

[30] Nguyen HA, Ahmed S, Turner HC (2023). Overview of the main methods used for estimating catastrophic health expenditure. Cost Eff Resour Alloc.

[31] Laokri S, Soelaeman R, Hotchkiss DR (2018). Assessing out-of-pocket expenditures for primary health care: how responsive is the Democratic Republic of Congo health system to providing financial risk protection?. BMC Health Serv Res.

[32] Wang W, Temsah G, Carter E (2016). Levels and determinants of out-of-pocket health expenditures in the Democratic Republic of the Congo, liberia, Namibia, and Rwanda. DHS analytical studies no 59.

[33] Brunner B, Combet V, Callahan S (2018). The role of the private sector in improving the performance of the health system in the Democratic Republic of Congo.

[34] European Asylum Support Office (2021). Medical country of origin information report: Democratic Republic of Congo (DRC).

[35] Simarro PP, Cecchi G, Franco JR (2014). Mapping the capacities of fixed health facilities to cover people at risk of gambiense human African trypanosomiasis. Int J Health Geogr.

[36] (2024). Guidelines for the treatment of human African trypanosomiasis.

[37] Betu Kumeso VK, Kalonji WM, Rembry S (2023). Efficacy and safety of acoziborole in patients with human African trypanosomiasis caused by Trypanosoma brucei gambiense: a multicentre, open-label, single-arm, phase 2/3 trial. Lancet Infect Dis.

[38] (2019). WHO interim guidelines for the treatment of gambiense human African trypanosomiasis.

[39] Nicco E, Lejon V, Mwamba Miaka E (2025). The STROGHAT study protocol: An intervention study to evaluate safety, effectiveness and feasibility of treating gambiense HAT seropositive subjects with acoziborole. *Open Res Eur*.

[40] Inocencio da Luz R, Phanzu DM, Kiabanzawoko ON (2021). Feasibility of a dried blood spot strategy for serological screening and surveillance to monitor elimination of Human African Trypanosomiasis in the Democratic Republic of the Congo. PLoS Negl Trop Dis.

