# Supplementary material for: The household burden of sleeping sickness: out-of-pocket costs for diagnosis and treatment
Source: BMJ Glob Health. 2026 Oct 16;11(10):e025040. doi: 10.1136/bmjgh-2026-025040 (PMC13691789; doi:10.1136/bmjgh-2026-025040)
Supplement: Supplementary file 1 [file bmjgh-11-10-s001.docx]

I. **Supplementary information**

[Supporting Information 1 : Number of new reported gHAT cases during the last 10 years^1^	3](#_Toc235524301)

[Supporting Information 2 : Map of the study area^2^	3](#_Toc235524302)

[Supporting Information 3 : Table: Composition of FGDs and SSIs for each health zone	3](#_Toc235524303)

[Supporting Information 4 : Table Knowledge on HAT characteristics by screening strategy	4](#_Toc235524304)

[Supporting Information 5: Bar chart Awareness of HAT cases among participants by Screening Strategy	4](#_Toc235524305)

[Supporting Information 6: Bar charts Reported symptoms prior to HAT by Screening Strategy	5](#_Toc235524306)

[Supporting Information 7: Table Health Care Seeking Behavior Pathway of participants experiencing symptoms prior to screening	6](#_Toc235524307)

[Supporting Information 8: Table Visits reported and travel time per type of health care structure	6](#_Toc235524308)

[Supporting Information 9: Table Means of travel used for screening, confirmation and treatment by HAT screening outcome	7](#_Toc235524309)

[Supporting Information 10: Table Travel time for screening, confirmation and treatment by HAT screening outcome	8](#_Toc235524310)

[Supporting Information 11: Reported revenue loss due to screening, microscopy and treatment	9](#_Toc235524311)

[Supporting Information 12: Summary of Statistical Tests	10](#_Toc235524312)

[Supporting Information 13: GLM regression Analysis of the Probability of Incurring Out-of-Pocket Costs by visit	12](#_Toc235524313)

[Supporting Information 14: GLM regression Analysis of the level of Out-of-Pocket Costs by visits reporting OOP	13](#_Toc235524314)

[Supporting Information 15: Table Median OOP per cost type per type of health structure	16](#_Toc235524315)

[Supporting Information 16: Table Median OOP per screening strategy per screening outcome	19](#_Toc235524316)

[Supporting Information 17: Frequency of the structures visited by participants	23](#_Toc235524317)

[Supporting Information 18: Interview guide – Individual semi-structured interviews	24](#_Toc235524318)

[Supporting Information 19: Interview guide – Focus group discussions	26](#_Toc235524319)

[Supporting Information 20: Survey « Evaluation des dépenses médicales et non-médicales THA »	27](#_Toc235524320)

[Supporting Information 21: GRAMMS compliance table	38](#_Toc235524321)

References ……………………………………………………………………………………………………………….38

**Supporting Information 1 : Number of new reported gHAT cases during the last 10 years^1^**

**Supporting Information 2 : Map of the study area^2^**

**Supporting Information 3 : Table: Composition of FGDs and SSIs for each health zone**

| **Participants FGD’s and SSIs** | **HZ A** | **HZ B** |
| --- | --- | --- |
| FGD Mixed: Health facility workers working in facilities without HAT screening |  | 1 |
| FGD Mixed: Health facility workers working in facilities with HAT screening |  | 1 |
| FGD Male, HAT endemic villages | 1 | 1 |
| FGD Female, HAT endemic villages | 1 | 1 |
| SSI Community Leader Male, HAT endemic villages | 1 | 1 |
| SSI PS Male and Female, HAT endemic villages | 6 | 1 |
| SSI AS Male and Female, HAT endemic village | 2 | 5 |
| **Total** | **11** | **11** |

Supporting Information 4 : Table Knowledge on HAT characteristics by screening strategy

|  | **Active** | **Passive** | **Overall** |
| --- | --- | --- | --- |
|  | **(N=230)** | **(N=214)** | **(N=444)** |
| **Knowledge on HAT** |  |  |  |
| Mean (SD) | 2.87 (1.10) | 2.97 (1.23) | 2.91 (1.16) |
| Median [Min, Max] | 3.00 [0, 5.00] | 3.00 [0, 5.00] | 3.00 [0, 5.00] |
| **HAT characteristics reported** |  |  |  |
| Sleep disorders | 148 (64.3%) | 135 (63.1%) | 283 (63.7%) |
| Headaches | 134 (58.3%) | 128 (59.8%) | 262 (59.0%) |
| Fever | 115 (50.0%) | 107 (50.0%) | 222 (50.0%) |
| Fatigue | 68 (29.6%) | 59 (27.6%) | 127 (28.6%) |
| Muscle and joint pain | 43 (18.7%) | 34 (15.9%) | 77 (17.3%) |
| Behavioral disorders | 32 (13.9%) | 39 (18.2%) | 71 (16.0%) |
| Other | 24 (10.4%) | 40 (18.7%) | 64 (14.4%) |
| Tse tse flies | 31 (13.5%) | 19 (8.9%) | 50 (11.3%) |
| Pruritis, itching | 26 (11.3%) | 14 (6.5%) | 40 (9.0%) |
| Loss of appetite and/or weight loss | 11 (4.8%) | 16 (7.5%) | 27 (6.1%) |
| Neurological disorders | 8 (3.5%) | 15 (7.0%) | 23 (5.2%) |
| Nothing | 9 (3.9%) | 8 (3.7%) | 17 (3.8%) |
| A fatal disease | 2 (0.9%) | 13 (6.1%) | 15 (3.4%) |
| Vomiting, dizziness, nausea, diarrhea | 4 (1.7%) | 7 (3.3%) | 11 (2.5%) |
| Low back pain, lumbar pain | 3 (1.3%) | 6 (2.8%) | 9 (2.0%) |
| Loss of consciousness, coma | 6 (2.6%) | 3 (1.4%) | 9 (2.0%) |
| Ganglions | 4 (1.7%) | 0 (0%) | 4 (0.9%) |

Supporting Information 5: Bar chart Awareness of HAT cases among participants by Screening Strategy

Supporting Information 6: Bar charts Reported symptoms prior to HAT by Screening Strategy

Supporting Information 7: Table Health Care Seeking Behavior Pathway of participants experiencing symptoms prior to screening

| **Type of health structure** | **1St** | **2nd** | **3rd** | **4th** | **5th** | **6th** | **7th** | **8th** | **9th** |
| --- | --- | --- | --- | --- | --- | --- | --- | --- | --- |
| Traditional practitioner | 5(3%) | 57(33%) | 79(45%) | 19(11%) | 10(6%) | 2(1%) | 2(1%) | 0(0%) | 0(0%) |
| Pharmacy | 71(41%) | 4(2%) | 58(33%) | 38(22%) | 3(2%) | 0(0%) | 0(0%) | 0(0%) | 0(0%) |
| Primary care facility | 79(45%) | 41(24%) | 9(5%) | 41(24%) | 3(2%) | 1(1%) | 0(0%) | 0(0%) | 0(0%) |
| Hospital | 16(9%) | 58(33%) | 20(11%) | 62(36%) | 17(10%) | 1(1%) | 0(0%) | 0(0%) | 0(0%) |
| Private medical practices | 2(1%) | 3(2%) | 0(0%) | 1(1%) | 139(80%) | 27(16%) | 2(1%) | 0(0%) | 0(0%) |
| Mobile team with a vehicle | 1(1%) | 7(4%) | 4(2%) | 10(6%) | 1(1%) | 142(82%) | 9(5%) | 0(0%) | 0(0%) |
| Mobile team with motorcycles | 0(0%) | 2(1%) | 2(1%) | 1(1%) | 1(1%) | 1(1%) | 161(93%) | 6(3%) | 0(0%) |
| Screening, diagnosis, treatment, and control center (CDTC) | 0(0%) | 2(1%) | 1(1%) | 2(1%) | 0(0%) | 0(0%) | 0(0%) | 168(97%) | 1(1%) |
| Other | 0(0%) | 0(0%) | 1(1%) | 0(0%) | 0(0%) | 0(0%) | 0(0%) | 0(0%) | 173(99%) |

Supporting Information 8: Table Visits reported and travel time per type of health care structure

| **Structure type** | **Traveltime** | **Frequency** | **%** |
| --- | --- | --- | --- |
| Pharmacy | 0 | 12 | 12% |
| Pharmacy | 1hr or less | 88 | 87% |
| Pharmacy | half a day or less | 1 | 1% |
| **Pharmacy Total** |  | **101** | **100%** |
| Health post - Health center - Referral center | 0 | 39 | 14% |
| Health post - Health center - Referral center | 1hr or less | 160 | 57% |
| Health post - Health center - Referral center | half a day or less | 73 | 26% |
| Health post - Health center - Referral center | 1 day | 7 | 3% |
| Health post - Health center - Referral center | 2 days | 1 | 0% |
| **Health post - Health center - Referral center Total** |  | **280** | **100%** |
| Secondary hospital - General referral hospital | 0 | 4 | 2% |
| Secondary hospital - General referral hospital | 1hr or less | 78 | 48% |
| Secondary hospital - General referral hospital | half a day or less | 56 | 35% |
| Secondary hospital - General referral hospital | 1 day | 22 | 14% |
| Secondary hospital - General referral hospital | 2 days | 1 | 1% |
| **Secondary hospital - General referral hospital Total** |  | **161** | **100%** |
| A mobile team with a vehicle | 0 | 88 | 47% |
| A mobile team with a vehicle | 1hr or less | 92 | 49% |
| A mobile team with a vehicle | half a day or less | 8 | 4% |
| A mobile team with a vehicle | 1 day | 1 | 1% |
| **A mobile team with a vehicle Total** |  | **189** | **100%** |
| A mobile team with motorcycles | 0 | 15 | 37% |
| A mobile team with motorcycles | 1hr or less | 25 | 61% |
| A mobile team with motorcycles | half a day or less | 1 | 2% |
| **A mobile team with motorcycles Total** |  | **41** | **100%** |
| Other | 0 | 2 | 100% |
| **Other Total** |  | **2** | **100%** |
| Private center - Private medical practice | 0 | 1 | 50% |
| Private center - Private medical practice | half a day or less | 1 | 50% |
| **Private center - Private medical practice Total** |  | **2** | **100%** |
| Traditional practitioners | 1hr or less | 5 | 45% |
| Traditional practitioners | 1 day | 5 | 45% |
| Traditional practitioners | 2 days | 1 | 9% |
| **Traditional practitioners Total** |  | **11** | **100%** |
| Screening, diagnosis, treatment, and monitoring center (CDTC) | half a day or less | 5 | 38% |
| Screening, diagnosis, treatment, and monitoring center (CDTC) | 1hr or less | 6 | 46% |
| Screening, diagnosis, treatment, and monitoring center (CDTC) | 1 day | 1 | 8% |
| Screening, diagnosis, treatment, and monitoring center (CDTC) | 2 days | 1 | 8% |
| **Screening, diagnosis, treatment, and monitoring center (CDTC) Total** |  | **13** | **100%** |
| **Grand Total** |  | **800** |  |

Supporting Information 9: Table Means of travel used for screening, confirmation and treatment by HAT screening outcome

| **Means of travel** | **Serology negative** | | | **Serology result unknown** | | | **Serology positive with microscopy not done/unknown** | | | **Serology performed with microscopy negative** | | | **Confirmed HAT case** | | | **Overall** | | |
| --- | --- | --- | --- | --- | --- | --- | --- | --- | --- | --- | --- | --- | --- | --- | --- | --- | --- | --- |
|  | **AS** | **PS** | **Total** | **AS** | **PS** | **Total** | **AS** | **PS** | **Total** | **AS** | **PS** | **Total** | **AS** | **PS** | **Total** | **AS** | **PS** | **Total** |
| **Screening** | **(N=128)** | **(N=134)** | **(N=262)** | **(N=10)** | **(N=11)** | **(N=21)** | **(N=13)** | **(N=9)** | **(N=22)** | **(N=61)** | **(N=32)** | **(N=93)** | **(N=18)** | **(N=28)** | **(N=46)** | **(N=230)** | **(N=214)** | **(N=444)** |
| Screened without travel | 103 (80.5%) | 13 (9.7%) | 116 (44.3%) | 10 (100%) | 1  (9.1%) | 11 (52.4%) | 13 (100%) | 3 (33.3%) | 16 (72.7%) | 46 (75.4%) | 15 (46.9%) | 61 (65.6%) | 17 (94.4%) | 10 (35.7%) | 27 (58.7%) | 189 (82.2%) | 42 (19.6%) | 231 (52.0%) |
| On foot | 19 (14.8%) | 81 (60.4%) | 100 (38.2%) | 0  (0%) | 6 (54.5%) | 6 (28.6%) | 0  (0%) | 5 (55.6%) | 5 (22.7%) | 15 (24.6%) | 12 (37.5%) | 27 (29.0%) | 1  (5.6%) | 16 (57.1%) | 17 (37.0%) | 35 (15.2%) | 120 (56.1%) | 155 (34.9%) |
| By bike | 1  (0.8%) | 8  (6.0%) | 9  (3.4%) | 0  (0%) | 0  (0%) | 0  (0%) | 0  (0%) | 1 (11.1%) | 1  (4.5%) | 0  (0%) | 1  (3.1%) | 1  (1.1%) | 0  (0%) | 0  (0%) | 0 (0%) | 1  (0.4%) | 10 (4.7%) | 11 (2.5%) |
| By motorcycle | 3  (2.3%) | 31 (23.1%) | 34 (13.0%) | 0  (0%) | 4 (36.4%) | 4 (19.0%) | 0  (0%) | 0  (0%) | 0  (0%) | 0  (0%) | 4 (12.5%) | 4  (4.3%) | 0  (0%) | 2  (7.1%) | 2  (4.3%) | 3  (1.3%) | 41 (19.2%) | 44 (9.9%) |
| Other^[1]^ | 2  (1.6%) | 1  (0.7%) | 3  (1.1%) | 0  (0%) | 0  (0%) | 0  (0%) | 0  (0%) | 0  (0%) | 0  (0%) | 0  (0%) | 0  (0%) | 0  (0%) | 0  (0%) | 0  (0%) | 0  (0%) | 2  (0.9%) | 1 (0.5%) | 3  (0.7%) |
| **Confirmation** |  |  |  |  |  |  |  |  |  | **(N=61)** | **(N=32)** | **(N=93)** | **(N=18)** | **(N=28)** | **(N=46)** | **(N=79)** | **(N=60)** | **(N=139)** |
| On the spot follow-up testing |  |  |  |  |  |  |  |  |  | 54 (88.5%) | 26 (81.3%) | 80 (86.0%) | 15 (83.3%) | 26 (92.9%) | 41 (89.1%) | 69 (87.3%) | 52 (86.7%) | 121 (87.0%) |
| Follow-up testing later at home |  |  |  |  |  |  |  |  |  | 1  (1.6%) | 6 (18.8%) | 7  (7.5%) | 1  (5.6%) | 0  (0%) | 1  (2.2%) | 2  (2.5%) | 6 (10,0%) | 8  (5.7%) |
| On foot |  |  |  |  |  |  |  |  |  | 5  (8.2%) | 0  (0%) | 5  (5.4%) | 1  (5.6%) | 2  (7.1%) | 3  (6.5%) | 6  (7.6%) | 2  (3,3%) | 8  (5.7%) |
| By motorcycle |  |  |  |  |  |  |  |  |  | 1  (1.6%) | 0  (0%) | 1  (1.1%) | 1  (5.6%) | 0  (0%) | 1  (2.2%) | 2  (2.5%) | 0  (0%) | 2  (1.6%) |
| **Treatment** |  |  |  |  |  |  |  |  |  |  |  |  | **(N=18)** | **(N=28)** | **(N=46)** | **(N=18)** | **(N=28)** | **(N=46)** |
| Treated without travel |  |  |  |  |  |  |  |  |  |  |  |  | 4 (22.2%) | 16 (57.1%) | 20 (43.5%) | 4 (22.2%) | 16 (57.1%) | 20 (43.5%) |
| On foot |  |  |  |  |  |  |  |  |  |  |  |  | 9 (50.0%) | 8 (28.6%) | 17 (37.0%) | 9 (50.0%) | 8 (28.6%) | 17 (37.0%) |
| By motorcycle |  |  |  |  |  |  |  |  |  |  |  |  | 4 (22.2%) | 3 (10.7%) | 7 (15.2%) | 4 (22.2%) | 3 (10.7%) | 7 (15.2%) |
| By car |  |  |  |  |  |  |  |  |  |  |  |  | 1  (5.6%) | 0  (0%) | 1  (2.2%) | 1  (5.6%) | 0  (0%) | 1  (2.2%) |
| Other |  |  |  |  |  |  |  |  |  |  |  |  | 0  (0%) | 1  (3.6%) | 1  (2.2%) | 0  (0%) | 1  (3.6%) | 1  (2.2%) |

Supporting Information 10: Table Travel time for screening, confirmation and treatment by HAT screening outcome

| **Travel time** | **Serology negative** | | | **Serology result unknown** | | | **Serology positive with microscopy not done/unknown** | | | **Serology performed with microscopy negative** | | | **Confirmed HAT case** | | | **Overall** | | |
| --- | --- | --- | --- | --- | --- | --- | --- | --- | --- | --- | --- | --- | --- | --- | --- | --- | --- | --- |
|  | **AS** | **PS** | **Total** | **AS** | **PS** | **Total** | **AS** | **PS** | **Total** | **AS** | **PS** | **Total** | **AS** | **PS** | **Total** | **AS** | **PS** | **Total** |
| **Screening** | **(N=128)** | **(N=134)** | **(N=262)** | **(N=10)** | **(N=11)** | **(N=21)** | **(N=10)** | **(N=11)** | **(N=21)** | **(N=61)** | **(N=32)** | **(N=93)** | **(N=18)** | **(N=28)** | **(N=46)** | **(N=230)** | **(N=214)** | **(N=444)** |
| No travel time | 103 (80.5%) | 13 (9.7%) | 116 (44.3%) | 10 (100%) | 1 (9.1%) | 11 (52.4%) | 13 (100%) | 3 (33.3%) | 16 (72.7%) | 46 (75.4%) | 15 (46.9%) | 61 (65.6%) | 17 (94.4%) | 10 (35.7%) | 27 (58.7%) | 189 (82.2%) | 42 (19.6%) | 231 (52.0%) |
| Less than 30 minutes | 14 (10.9%) | 63 (47.0%) | 77 (29.4%) | 0 (0%) | 5 (45.5%) | 5 (23.8%) | 0 (0%) | 4 (44.4%) | 4 (18.2%) | 8 (13.1%) | 9 (28.1%) | 17 (18.3%) | 1 (5.6%) | 4 (14.3%) | 5 (10.9%) | 23 (10.0%) | 85 (39.7%) | 108 (24.3%) |
| Less than an hour | 2  (1.6%) | 31 (23.1%) | 33 (12.6%) | 0 (0%) | 4 (36.4%) | 4 (19.0%) | 0 (0%) | 1 (11.1%) | 1 (4.5%) | 5 (8.2%) | 5 (15.6%) | 10 (10.8%) | 0 (0%) | 4 (14.3%) | 4 (8.7%) | 7 (3.0%) | 45 (21.0%) | 52 (11.7%) |
| Less than 2 hours | 4  (3.1%) | 15 (11.2%) | 19 (7.3%) | 0 (0%) | 1 (9.1%) | 1 (4.8%) | 0 (0%) | 0 (0%) | 0 (0%) | 2 (3.3%) | 0 (0%) | 2 (2.2%) | 0 (0%) | 3 (10.7%) | 3 (6.5%) | 6 (2.6%) | 19 (8.9%) | 25 (5.6%) |
| Less than 4 hours | 2  (1.6%) | 8  (6.0%) | 10 (3.8%) | 0 (0%) | 0 (0%) | 0 (0%) | 0 (0%) | 0 (0%) | 0 (0%) | 0 (0%) | 3 (9.4%) | 3 (3.2%) | 0 (0%) | 5 (17.9%) | 5 (10.9%) | 2 (0.9%) | 16 (7.5%) | 18 (4.1%) |
| Less than 8 hours | 2  (1.6%) | 3  (2.2%) | 5 (1.9%) | 0 (0%) | 0 (0%) | 0 (0%) | 0 (0%) | 1 (11.1%) | 1 (4.5%) | 0 (0%) | 0 (0%) | 0 (0%) | 0 (0%) | 1 (3.6%) | 1 (2.2%) | 2 (0.9%) | 5 (2.3%) | 7 (1.6%) |
| Less than a day | 1  (0.8%) | 1  (0.7%) | 2 (0.8%) | 0 (0%) | 0 (0%) | 0 (0%) | 0 (0%) | 0 (0%) | 0 (0%) | 0 (0%) | 0 (0%) | 0 (0%) | 0 (0%) | 0 (0%) | 0 (0%) | 1 (0.4%) | 1 (0.5%) | 2 (0.5%) |
| More than one day, I had to spend the night somewhere along the way. | 0  (0%) | 0  (0%) | 0 (0%) | 0 (0%) | 0 (0%) | 0 (0%) | 0 (0%) | 0 (0%) | 0 (0%) | 0 (0%) | 0 (0%) | 0 (0%) | 0 (0%) | 1 (3.6%) | 1 (2.2%) | 0 (0%) | 1 (0.5%) | 1 (0.2%) |
| **Confirmation** |  |  |  |  |  |  |  |  |  | **(N=61)** | **(N=32)** | **(N=93)** | **(N=18)** | **(N=28)** | **(N=46)** | **(N=79)** | **(N=60)** | **(N=139)** |
| No additional travel required |  |  |  |  |  |  |  |  |  | 55 (90.2%) | 32 (100%) | 87 (93.5%) | 16 (88.9%) | 26 (92.9%) | 42 (91.3%) | 71 (89.9%) | 58 (96.7%) | 129 (92.8%) |
| Less than 30 minutes |  |  |  |  |  |  |  |  |  | 4 (6.6%) | 0 (0%) | 4 (4.3%) | 0 (0%) | 0 (0%) | 0 (0%) | 4 (5.1%) |  | 4 (2.9%) |
| Less than an hour |  |  |  |  |  |  |  |  |  | 1 (1.6%) | 0 (0%) | 1 (1.1%) | 0 (0%) | 0 (0%) | 0 (0%) | 1 (1.2%) |  | 1 (0.7%) |
| Less than 2 hours |  |  |  |  |  |  |  |  |  | 1 (1.6%) | 0 (0%) | 1 (1.1%) | 0 (0%) | 0 (0%) | 0 (0%) | 1 (1.2%) |  | 1 (0.7%) |
| Less than 4 hours |  |  |  |  |  |  |  |  |  | 0 (0%) | 0 (0%) | 0 (0%) | 1 (5.6%) | 1 (3.6%) | 2 (4.3%) | 1 (1.2%) | 1 (1.6%) | 2 (1.4%) |
| Less than 8 hours |  |  |  |  |  |  |  |  |  | 0 (0%) | 0 (0%) | 0 (0%) | 1 (5.6%) | 1 (3.6%) | 2 (4.3%) | 1 (1.2%) | 1 (1.6%) | 2 (1.4%) |
| **Treatment** |  |  |  |  |  |  |  |  |  |  |  |  | **(N=18)** | **(N=28)** | **(N=46)** | **(N=18)** | **(N=28)** | **(N=46)** |
| No travel time |  |  |  |  |  |  |  |  |  |  |  |  | 4 (22.2%) | 16 (57.1%) | 20 (43.5%) | 4 (22.2%) | 16 (57.1%) | 20 (43.5%) |
| Less than 30 minutes |  |  |  |  |  |  |  |  |  |  |  |  | 0 (0%) | 3 (10.7%) | 3 (6.5%) | 0 (0%) | 3 (10.7%) | 3 (6.5%) |
| Less than an hour |  |  |  |  |  |  |  |  |  |  |  |  | 3 (16.7%) | 1 (3.6%) | 4 (8.7%) | 3 (16.7%) | 1 (3.6%) | 4 (8.7%) |
| Less than 2 hours |  |  |  |  |  |  |  |  |  |  |  |  | 2 (11.1%) | 1 (3.6%) | 3 (6.5%) | 2 (11.1%) | 1 (3.6%) | 3 (6.5%) |
| Less than 4 hours |  |  |  |  |  |  |  |  |  |  |  |  | 4 (22.2%) | 3 (10.7%) | 7 (15.2%) | 4 (22.2%) | 3 (10.7%) | 7 (15.2%) |
| Less than 8 hours |  |  |  |  |  |  |  |  |  |  |  |  | 3 (16.7%) | 2 (7.1%) | 5 (10.9%) | 3 (16.7%) | 2 (7.1%) | 5 (10.9%) |
| Less than a day |  |  |  |  |  |  |  |  |  |  |  |  | 2 (11.1%) | 1 (3.6%) | 3 (6.5%) | 2 (11.1%) | 1 (3.6%) | 3 (6.5%) |
| More than one day, I had to spend the night somewhere along the way. |  |  |  |  |  |  |  |  |  |  |  |  | 0 (0%) | 1 (3.6%) | 1 (2.2%) | 0 (0%) | 1 (3.6%) | 1 (2.2%) |

Supporting Information 11: Reported revenue loss due to screening, microscopy and treatment

| **Revenue loss due to screening** | **Participants** | **% by Total** |
| --- | --- | --- |
| **No** | **203** | **46%** |
| Active | 148 | 33% |
| Passive | 55 | 12% |
| **Yes** | **241** | **54%** |
| Active | 82 | 18% |
| Passive | 159 | 36% |
| **Grand Total** | **444** | **100%** |
|  |  |  |
|  |  |  |
| **Revenue loss due to microscopy confirmation** | **Participants** | **% by Total** |
| **No** | **93** | **64%** |
| Active | 68 | 47% |
| Passive | 25 | 17% |
| **Yes** | **53** | **36%** |
| Active | 18 | 12% |
| Passive | 35 | 24% |
| **Grand Total** | **146** | **100%** |
|  |  |  |
|  |  |  |
|  |  |  |
| **Revenue loss due to treatment** | **Participants** | **% by Total** |
| **No** | **5** | **11%** |
| Active | 3 | 7% |
| Passive | 2 | 4% |
| **Yes** | **41** | **89%** |
| Active | 15 | 33% |
| Passive | 26 | 57% |
| **Grand Total** | **46** | **100%** |

Supporting Information 12: Summary of Statistical Tests

1. Comparison Positive and Zero-OOP Proportions Between Screening strategies for visits and participants, including food costs

|  |  |  | **Visits (n= 800)** | | | | | | **Participants (n=444)** | | | | | |
| --- | --- | --- | --- | --- | --- | --- | --- | --- | --- | --- | --- | --- | --- | --- |
| **Strategy** |  | **Comparison** | **n**  **OOP=0** | **n**  **OOP >0** | **test** | **Chi-square** | **df** | **p_value** | **n**  **OOP=0** | **n**  **OOP >0** | **test** | **Chi-square** | **df** | **p_value** |
| AS |  | Zero OOP vs Positive OOP | 215 | 15 | Chi-square | 467.58 | 1 | p < 0.0001 | 126 | 104 | Chi-square | 83.25 | 1 | p < 0.0001 |
| PS |  | Zero OOP vs Positive OOP | 70 | 500 |  |  |  |  | 28 | 186 | Chi-square |  |  |  |
|  |  |  | **285** | **515** |  |  |  |  | 154 | 290 |  |  |  |  |

2. Comparison Positive and Zero-OOP Proportions Between Screening strategies for visits and participants, excluding food costs

|  |  |  | **Visits (n= 800)** | | | | | | **Participants (n=444)** | | | | | |
| --- | --- | --- | --- | --- | --- | --- | --- | --- | --- | --- | --- | --- | --- | --- |
| **Strategy** |  | **Comparison** | **n**  **OOP=0** | **n**  **OOP >0** | **test** | **Chi-square** | **df** | **p_value** | **n**  **OOP=0** | **n**  **OOP >0** | **test** | **Chi-square** | **df** | **p_value** |
| AS |  | Zero OOP vs Positive OOP | 223 | 7 | Chi-square | 449.89 | 1 | p < 0.0001 | 134 | 96 | Chi-square | 76.86 | 1 | p < 0.0001 |
| PS |  | Zero OOP vs Positive OOP | 90 | 480 |  |  |  |  | 37 | 177 | Chi-square |  |  |  |
|  |  |  | **313** | **487** |  |  |  |  | 171 | 273 |  |  |  |  |

3. Comparison of Median OOP Costs Between Screening Strategies Among Visits With Positive OOP, including food costs

|  |  | **Visits (n= 800)** | | | | | **Participants (n=444)** | | | | |
| --- | --- | --- | --- | --- | --- | --- | --- | --- | --- | --- | --- |
|  | **Comparison** | **AS** | **PS** | **test** | **MW** | **p_value** | **AS** | **PS** | **test** | **MW** | **p_value** |
|  | Median OOP AS & PS including food costs | 0.76 | 9.08 | Mann-Whitney U-test | 966 | p < 0.0001 | 12.14 | 20.51 | Mann-Whitney U-test | 8324.5 | 0.05 |
|  | Median OOP AS & PS excluding food costs | 1.01 | 7.57 | Mann-Whitney U-test | 342.5 | P<0.001 | 10.80 | 17.66 | Mann-Whitney U-test | 7509 | 0.11 |

4. Comparison among confirmed cases of patients that were identified via AS vs PS, including food costs

|  |  |  | **Participants (n= 46)** | | | | | |
| --- | --- | --- | --- | --- | --- | --- | --- | --- |
| **Strategy** |  | **Comparison** | **n**  **OOP=0** | **n**  **OOP >0** | **test** | **p_value** | **95% CI** | **Odds ratio** |
| AS |  | Zero OOP vs Positive OOP | 4 | 14 | Fisher’s exact test | p = 0.019 | (1.13-Inf) | inf |
| PS |  | Zero OOP vs Positive OOP | 0 | 28 |  |  |  |  |
|  |  |  | **4** | **42** |  |  |  |  |

5. Comparison among confirmed cases of patients that were identified via AS vs PS, excluding food costs

|  |  |  | **Participants (n= 46)** | | | | | |
| --- | --- | --- | --- | --- | --- | --- | --- | --- |
| **Strategy** |  | **Comparison** | **n**  **OOP=0** | **n**  **OOP >0** | **test** | **p_value** | **95% CI** | **Odds ratio** |
| AS |  | Zero OOP vs Positive OOP | 7 | 11 | Fisher’s exact test | P=0.018 | (1.239 - 89.391) | 7.857 |
| PS |  | Zero OOP vs Positive OOP | 2 | 26 |  |  |  |  |
|  |  |  | **9** | **37** |  |  |  |  |

6. Comparison of Median OOP Costs Between Screening Strategies Among Confirmed Cases with Positive OOP

|  |  | **Participants (n=46)** | | | | |
| --- | --- | --- | --- | --- | --- | --- |
|  | **Comparison** | **AS (median)** | **PS (median)** | **test** | **MW** | **p_value** |
|  | Median OOP AS & PS including food costs | 9.84 | 24.52 | Mann-Whitney U-test | 92 | 0.006 |
|  | Median OOP AS & PS excluding food costs | 5.05 | 22.83 | Mann-Whitney U-test | 57 | 0.004 |

Supporting Information 13: GLM regression Analysis of the Probability of Incurring Out-of-Pocket Costs by visit

**Model:** Binomial logistic regression (N =800)
**Outcome variable**: No OOP versus OOP

**Predictor variables:** Type of health structure, Travel time, mode of transport, Screening outcome

| **Predictor** | **df** | **Deviance** | **AIC** | **LRT** | **p-value** |  |
| --- | --- | --- | --- | --- | --- | --- |
| **Full model** | — | 449.54 | 473.54 | — | — |  |
| Type of health structure | 4 | 848.51 | 864.51 | 398.97 | <0.00001 |  |
| Travel time | 2 | 455.26 | 475.26 | 5.73 | 0.057 |  |
| Mode of transport | 1 | 450.23 | 472.23 | 0.69 | 0.405 |  |
| Screening outcome | 4 | 454.43 | 470.43 | 4.90 | 0.298 |  |

Table1: Results Likelihood Ratio Tests for single-term deletions

Table2: Adjusted odds ratios (OR) and 95% confidence intervals (CI) for predictors of out-of-pocket (excluding screening outcome and mode of transport)

| **Predictor** | **OR** | **95% CI** | **p-value** | **Comment** |
| --- | --- | --- | --- | --- |
| **Health structure type  (ref: Mobile team: Vehicle or motorcycle)** |  |  |  |  |
| Pharmacy | 442.23 | 124.33-1572.63 | <0.001 | Strongest predictor |
| Primary care facility | 42.63 | 23.05-78.77 | <0.001 | Large effect |
| Hospital | 241.82 | 84.73-690.03 | <0.001 | Very strong effect |
| Other types | 40.45 | 12.62-129.14 | <0.001 | Large effect |
| **Travel time (ref: No travel reported)** |  |  |  |  |
| 1 hour or less | 4.17 | 2.27-7.64 | <0.001 | Moderate increase in odds |
| More than 1 hour | 9.86 | 4.42-22.00 | <0.001 | Moderate increase in odds |

Table3: Model diagnostics

| **Diagnostic** | **Value** | **Interpretation** |
| --- | --- | --- |
| Convergence | Successful | Model converged |
| Maximum VIF | 1.04 | Negligible multicollinearity model. |
| Residual uniformity, p-value | 0.679 | No evidence of systematic residual misspecification |
| Dispersion test, p-value | 0.948 | No evidence of overdispersion or underdispersion. |
| Residual outlier test, p-value | 0.418 | No excess residual outliers |
| Separation | Not detected | No complete or quasi-complete separation; maximum-likelihood estimates were finite. |

Supporting Information 14: GLM regression Analysis of the level of Out-of-Pocket Costs by visits reporting OOP

**Model:** Gamma GLM with log link (N = 515)
**Outcome variable**: Total OOP costs including food costs (USD) among participants with OOP > 0
**Predictor variables:** Type of health structure, Travel time, mode of transport, Screening outcome

| **Predictor** | **df** | **Deviance** | **AIC** | **Scaled dev.** | **p-value** |  |
| --- | --- | --- | --- | --- | --- | --- |
| **Full model** | — | 524 | 3,697 |  |  |  |
| Type of health structure | 4 | 845 | 3,946 | 257.45 | <0.001 |  |
| Travel time | 2 | 524 | 3,693 | 0.45 | 0.796 |  |
| Mode of transport | 1 | 524 | 3,694 | 0.2 | 0.727 |  |
| Screening outcome | 4 | 548 | 3,408 | 19.28 | <0.001 |  |

Table1: Results Likelihood Ratio Tests for single-term deletions

Table2: Adjusted exponentiated coefficients (cost multipliers) from the reduced model and 95% confidence intervals for predictors of OOP cost level

**Model:** Gaussian GLM with log link (N = 515)
**Outcome variable**: Total OOP costs (USD) among participants with OOP > 0
**Predictor variables:** Type of health structure, Screening outcome

| **Predictor** | **Exp(β)** | **95% CI** | **p-value** | **Interpretation** |
| --- | --- | --- | --- | --- |
| **Health structure type (ref: Mobile team)^[2]^** |  |  |  |  |
| Pharmacy | 2.16 | 1.12–3.82 | 0.013 | Higher costs |
| Primary care facility | 4.59 | 2.42–7.91 | <0.001 | Higher costs |
| Hospital | 21.15 | 11.09–36.77 | <0.001 | Substantially costs |
| Other types | 10.32 | 4.78–21.52 | <0.001 | Higher costs |
| **Screening outcome (ref: serology negative)** | | |  |  |
| Serology result unknown | 0.68 | 0.43–1.14 | 0.116 | No significant difference |
| Serology positive; microscopy not done or unknown | 1.07 | 0.64–1.94 | 0.788 | No significant difference |
| Serology performed; microscopy negative | 0.70 | 0.52–0.96 | 0.024 | Lower costs |
| Confirmed HAT case | 1.60 | 1.22–2.13 | 0.001 | Higher costs |

Table3: Model diagnostics

| **Diagnostic** | **Reduced model** | **Interpretation** |
| --- | --- | --- |
| Convergence | Successful | Model converged successfully. |
| Maximum VIF | 1.06 | Negligible multicollinearity. |
| Residual uniformity, p-value | 0.134 | No evidence of systematic residual misspecification. |
| Dispersion test, p-value | 0.338 | No evidence of problematic residual dispersion. |
| Residual outlier test, p-value | 0.086 | No statistically significant excess of residual outliers. |

Supporting Information 15: Table Median OOP per cost type per type of health structure

| **StructTyp** | **variable** | **n_total** | **n_nonzero** | **Min_Value** | **Q1** | **Median** | **Q3** | **Max_Value** | **IQR** |
| --- | --- | --- | --- | --- | --- | --- | --- | --- | --- |
| A mobile team with a vehicle | Medical OOP | 189 | 4.00 | 0.50 | 0.88 | 1.26 | 2.40 | 5.05 | 1.51 |
| A mobile team with a vehicle | Medical OOP: Consumables | 2 | 2.00 | 1.01 | 2.02 | 3.03 | 4.04 | 5.05 | 2.02 |
| A mobile team with a vehicle | Medical OOP: Laboratory | 1 | 1.00 | 0.50 | 0.50 | 0.50 | 0.50 | 0.50 | - |
| A mobile team with a vehicle | Medical OOP: Medication | 1 | 1.00 | 1.51 | 1.51 | 1.51 | 1.51 | 1.51 | - |
| A mobile team with a vehicle | Non-Medical OOP | 189 | 10.00 | 0.10 | 0.25 | 0.25 | 0.95 | 10.09 | 0.69 |
| A mobile team with a vehicle | Non-Medical OOP: Food | 8 | 8.00 | 0.10 | 0.25 | 0.25 | 2.27 | 10.09 | 2.02 |
| A mobile team with a vehicle | Non-Medical OOP: Other | 2 | 2.00 | 0.25 | 0.44 | 0.63 | 0.82 | 1.01 | 0.38 |
| A mobile team with a vehicle | Non-Medical OOP: Transport | 1 | 1.00 | 0.20 | 0.20 | 0.20 | 0.20 | 0.20 | - |
| A mobile team with a vehicle | Total OOP | 189 | 14.00 | 0.10 | 0.25 | 0.63 | 1.39 | 10.09 | 1.14 |
| A mobile team with a vehicle | Total OOP excluding food related costs | 189 | 7.00 | 0.20 | 0.38 | 1.01 | 1.26 | 5.05 | 0.88 |
| A mobile team with motorcycles | Non-Medical OOP | 41 | 1.00 | 2.02 | 2.02 | 2.02 | 2.02 | 2.02 | - |
| A mobile team with motorcycles | Non-Medical OOP: Food | 1 | 1.00 | 2.02 | 2.02 | 2.02 | 2.02 | 2.02 | - |
| A mobile team with motorcycles | Total OOP | 41 | 1.00 | 2.02 | 2.02 | 2.02 | 2.02 | 2.02 | - |
| A mobile team with motorcycles | Total OOP excluding food related costs | 41 | - | - | - | - | - | - | - |
| Health post - Health center - Referral center | Medical OOP | 280 | 193.00 | 0.25 | 3.03 | 5.15 | 8.58 | 76.08 | 5.55 |
| Health post - Health center - Referral center | Medical OOP: Consumables | 183 | 183.00 | 0.50 | 2.02 | 3.53 | 5.05 | 32.80 | 3.03 |
| Health post - Health center - Referral center | Medical OOP: Hospitalization | 17 | 17.00 | 0.50 | 2.52 | 3.53 | 7.57 | 10.60 | 5.05 |
| Health post - Health center - Referral center | Medical OOP: Laboratory | 55 | 55.00 | 0.10 | 0.50 | 1.01 | 1.64 | 22.70 | 1.14 |
| Health post - Health center - Referral center | Medical OOP: Medication | 78 | 78.00 | 0.25 | 2.52 | 3.53 | 6.05 | 22.70 | 3.53 |
| Health post - Health center - Referral center | Medical OOP: Other | 10 | 10.00 | 0.10 | 0.25 | 0.30 | 0.50 | 50.45 | 0.25 |
| Health post - Health center - Referral center | Medical OOP: Other purchases | 5 | 5.00 | 0.76 | 0.76 | 1.01 | 12.61 | 50.45 | 11.86 |
| Health post - Health center - Referral center | Non-Medical OOP | 280 | 171.00 | 0.10 | 0.63 | 1.51 | 4.04 | 58.02 | 3.41 |
| Health post - Health center - Referral center | Non-Medical OOP: Accommodation | 2 | 2.00 | 1.26 | 2.71 | 4.16 | 5.61 | 7.06 | 2.90 |
| Health post - Health center - Referral center | Non-Medical OOP: Food | 140 | 140.00 | 0.10 | 0.73 | 1.51 | 3.09 | 50.45 | 2.36 |
| Health post - Health center - Referral center | Non-Medical OOP: Other | 22 | 22.00 | 0.25 | 0.25 | 0.63 | 2.31 | 15.14 | 2.06 |
| Health post - Health center - Referral center | Non-Medical OOP: Transport | 59 | 59.00 | 0.25 | 0.50 | 1.01 | 3.28 | 22.70 | 2.77 |
| Health post - Health center - Referral center | Total OOP | 280 | 223.00 | 0.25 | 3.36 | 6.56 | 11.00 | 128.05 | 7.64 |
| Health post - Health center - Referral center | Total OOP excluding food related costs | 280 | 210.00 | 0.25 | 3.03 | 5.32 | 9.04 | 77.60 | 6.02 |
| Other | Medical OOP | 2 | 1.00 | 2.52 | 2.52 | 2.52 | 2.52 | 2.52 | - |
| Other | Medical OOP: Consumables | 1 | 1.00 | 2.52 | 2.52 | 2.52 | 2.52 | 2.52 | - |
| Other | Total OOP | 2 | 1.00 | 2.52 | 2.52 | 2.52 | 2.52 | 2.52 | - |
| Other | Total OOP excluding food related costs | 2 | 1.00 | 2.52 | 2.52 | 2.52 | 2.52 | 2.52 | - |
| Other | Total OOP excluding food related costs | 2 | - | - | 0.63 | 1.26 | 1.89 | 3.78 | 2.52 |
| Pharmacy | Medical OOP | 101 | 97.00 | 0.25 | 1.01 | 2.52 | 5.05 | 25.23 | 4.04 |
| Pharmacy | Medical OOP: Consumables | 9 | 9.00 | 2.52 | 3.53 | 7.57 | 10.09 | 25.23 | 6.56 |
| Pharmacy | Medical OOP: Medication | 88 | 88.00 | 0.25 | 0.76 | 2.14 | 5.05 | 25.23 | 4.29 |
| Pharmacy | Non-Medical OOP | 101 | 3.00 | 3.53 | 4.29 | 5.05 | 6.31 | 7.57 | 2.02 |
| Pharmacy | Non-Medical OOP: Accommodation | 1 | 1.00 | 1.01 | 1.01 | 1.01 | 1.01 | 1.01 | - |
| Pharmacy | Non-Medical OOP: Food | 3 | 3.00 | 0.50 | 2.27 | 4.04 | 4.54 | 5.05 | 2.27 |
| Pharmacy | Non-Medical OOP: Transport | 2 | 2.00 | 2.52 | 2.65 | 2.77 | 2.90 | 3.03 | 0.25 |
| Pharmacy | Total OOP | 101 | 98.00 | 0.25 | 1.01 | 2.52 | 5.05 | 25.23 | 4.04 |
| Pharmacy | Total OOP excluding food related costs | 101 | 98.00 | 0.25 | 1.01 | 2.52 | 5.05 | 25.23 | 4.04 |
| Private center - Private medical practice | Medical OOP | 2 | 2.00 | 14.13 | 15.01 | 15.89 | 16.78 | 17.66 | 1.77 |
| Private center - Private medical practice | Medical OOP: Consumables | 2 | 2.00 | 5.05 | 6.31 | 7.57 | 8.83 | 10.09 | 2.52 |
| Private center - Private medical practice | Medical OOP: Laboratory | 1 | 1.00 | 2.52 | 2.52 | 2.52 | 2.52 | 2.52 | - |
| Private center - Private medical practice | Medical OOP: Medication | 2 | 2.00 | 6.56 | 6.81 | 7.06 | 7.32 | 7.57 | 0.50 |
| Private center - Private medical practice | Non-Medical OOP | 2 | 1.00 | 7.57 | 7.57 | 7.57 | 7.57 | 7.57 | - |
| Private center - Private medical practice | Non-Medical OOP: Food | 1 | 1.00 | 7.57 | 7.57 | 7.57 | 7.57 | 7.57 | - |
| Private center - Private medical practice | Total OOP | 2 | 2.00 | 17.66 | 18.67 | 19.68 | 20.69 | 21.70 | 2.02 |
| Private center - Private medical practice | Total OOP excluding food related costs | 2 | 2.00 | 14.13 | 15.01 | 15.89 | 16.78 | 17.66 | 1.77 |
| Private center - Private medical practice | Total OOP excluding food related costs | 2 | 14.13 | 12.36 | 15.01 | 15.89 | 16.78 | 19.42 | 17.66 |
| Screening, diagnosis, treatment, and monitoring center (CDTC) | Medical OOP | 13 | 6.00 | 6.05 | 6.12 | 6.94 | 8.32 | 10.09 | 2.21 |
| Screening, diagnosis, treatment, and monitoring center (CDTC) | Medical OOP: Consumables | 1 | 1.00 | 8.07 | 8.07 | 8.07 | 8.07 | 8.07 | - |
| Screening, diagnosis, treatment, and monitoring center (CDTC) | Medical OOP: Hospitalization | 4 | 4.00 | 6.05 | 6.24 | 6.94 | 8.20 | 10.09 | 1.96 |
| Screening, diagnosis, treatment, and monitoring center (CDTC) | Medical OOP: Laboratory | 1 | 1.00 | 0.50 | 0.50 | 0.50 | 0.50 | 0.50 | - |
| Screening, diagnosis, treatment, and monitoring center (CDTC) | Medical OOP: Medication | 1 | 1.00 | 6.05 | 6.05 | 6.05 | 6.05 | 6.05 | - |
| Screening, diagnosis, treatment, and monitoring center (CDTC) | Non-Medical OOP | 13 | 7.00 | 1.26 | 6.31 | 9.33 | 38.09 | 90.82 | 31.79 |
| Screening, diagnosis, treatment, and monitoring center (CDTC) | Non-Medical OOP: Food | 4 | 4.00 | 4.29 | 8.64 | 23.97 | 43.52 | 60.54 | 34.88 |
| Screening, diagnosis, treatment, and monitoring center (CDTC) | Non-Medical OOP: Other | 2 | 2.00 | 0.50 | 7.95 | 15.39 | 22.83 | 30.27 | 14.88 |
| Screening, diagnosis, treatment, and monitoring center (CDTC) | Non-Medical OOP: Transport | 5 | 5.00 | 1.26 | 5.05 | 6.05 | 6.56 | 27.75 | 1.51 |
| Screening, diagnosis, treatment, and monitoring center (CDTC) | Total OOP | 13 | 9.00 | 1.26 | 10.09 | 13.62 | 16.65 | 90.82 | 6.56 |
| Screening, diagnosis, treatment, and monitoring center (CDTC) | Total OOP excluding food related costs | 13 | 9.00 | 1.26 | 8.58 | 11.10 | 13.62 | 30.27 | 5.05 |
| Secondary hospital - General referral hospital | Medical OOP | 161 | 147.00 | 0.50 | 11.83 | 22.20 | 36.07 | 376.14 | 24.24 |
| Secondary hospital - General referral hospital | Medical OOP: Consumables | 141 | 141.00 | 0.76 | 2.27 | 6.05 | 13.62 | 85.77 | 11.35 |
| Secondary hospital - General referral hospital | Medical OOP: Hospitalization | 39 | 39.00 | 1.26 | 3.78 | 6.05 | 10.09 | 30.27 | 6.31 |
| Secondary hospital - General referral hospital | Medical OOP: Laboratory | 74 | 73.00 | 0.25 | 1.01 | 1.77 | 3.53 | 28.25 | 2.52 |
| Secondary hospital - General referral hospital | Medical OOP: Medication | 108 | 108.00 | 0.25 | 7.57 | 12.61 | 20.81 | 100.91 | 13.24 |
| Secondary hospital - General referral hospital | Medical OOP: Other | 39 | 39.00 | 0.35 | 1.01 | 3.03 | 4.79 | 17.66 | 3.78 |
| Secondary hospital - General referral hospital | Medical OOP: Other purchases | 5 | 5.00 | 1.01 | 6.05 | 30.27 | 75.68 | 343.09 | 69.63 |
| Secondary hospital - General referral hospital | Non-Medical OOP | 161 | 146.00 | 0.25 | 5.87 | 10.09 | 17.44 | 160.95 | 11.58 |
| Secondary hospital - General referral hospital | Non-Medical OOP: Accommodation | 14 | 14.00 | 2.52 | 5.11 | 7.06 | 9.84 | 15.14 | 4.73 |
| Secondary hospital - General referral hospital | Non-Medical OOP: Food | 134 | 134.00 | 0.25 | 5.05 | 9.08 | 12.61 | 100.91 | 7.57 |
| Secondary hospital - General referral hospital | Non-Medical OOP: Other | 22 | 22.00 | 0.25 | 0.76 | 1.14 | 4.67 | 151.36 | 3.91 |
| Secondary hospital - General referral hospital | Non-Medical OOP: Transport | 66 | 66.00 | 0.25 | 1.58 | 3.78 | 6.05 | 37.84 | 4.48 |
| Secondary hospital - General referral hospital | Total OOP | 161 | 156.00 | 0.50 | 17.02 | 33.68 | 53.73 | 409.18 | 36.72 |
| Secondary hospital - General referral hospital | Total OOP excluding food related costs | 161 | 149.00 | 0.76 | 12.87 | 24.22 | 42.13 | 383.96 | 29.26 |
| Traditional practitioners | Medical OOP | 11 | 11.00 | 1.77 | 19.42 | 22.70 | 29.01 | 35.32 | 9.59 |
| Traditional practitioners | Medical OOP: Consumables | 5 | 5.00 | 2.52 | 3.03 | 3.18 | 5.05 | 18.16 | 2.02 |
| Traditional practitioners | Medical OOP: Medication | 2 | 2.00 | 17.66 | 19.55 | 21.44 | 23.34 | 25.23 | 3.78 |
| Traditional practitioners | Medical OOP: Other | 8 | 8.00 | 1.77 | 17.41 | 23.46 | 25.86 | 35.32 | 8.45 |
| Traditional practitioners | Non-Medical OOP | 11 | 8.00 | 1.51 | 2.84 | 4.41 | 7.69 | 15.64 | 4.86 |
| Traditional practitioners | Non-Medical OOP: Food | 8 | 8.00 | 1.26 | 1.51 | 3.41 | 7.69 | 8.58 | 6.18 |
| Traditional practitioners | Non-Medical OOP: Transport | 3 | 3.00 | 1.51 | 2.02 | 2.52 | 4.79 | 7.06 | 2.77 |
| Traditional practitioners | Total OOP | 11 | 11.00 | 10.85 | 20.18 | 27.75 | 31.61 | 37.84 | 11.43 |
| Traditional practitioners | Total OOP excluding food related costs | 11 | 11.00 | 8.83 | 19.42 | 23.21 | 29.01 | 35.32 | 9.59 |

Supporting Information 16: Table Median OOP per screening strategy per screening outcome

| **ScreeningStrat** | **Outcome_PLNTHA** | **variable** | **n_total** | **n_nonzero** | **Min_Value** | **Q1** | **Median** | **Q3** | **Max_Value** | **IQR** |
| --- | --- | --- | --- | --- | --- | --- | --- | --- | --- | --- |
| Active | Serology: negative | Medical OOP: Consumables | 128 | 52 | 1.26 | 4.04 | 8.20 | 20.81 | 89.81 | 16.78 |
| Active | Serology: result unknown | Medical OOP: Consumables | 10 | 2 | 3.53 | 6.24 | 8.96 | 11.67 | 14.38 | 5.42 |
| Active | Serology: positive; Microscopy: not done/unknown | Medical OOP: Consumables | 13 | 2 | 2.52 | 4.41 | 6.31 | 8.20 | 10.09 | 3.78 |
| Active | Serology performed; Microscopy negative | Medical OOP: Consumables | 61 | 15 | 0.50 | 2.27 | 3.03 | 5.55 | 16.40 | 3.28 |
| Active | Confirmed HAT case | Medical OOP: Consumables | 18 | 6 | 0.76 | 0.95 | 1.51 | 2.27 | 5.55 | 1.32 |
| Passive | Serology: negative | Medical OOP: Consumables | 134 | 104 | 0.50 | 1.70 | 6.56 | 10.60 | 62.06 | 8.89 |
| Passive | Serology: result unknown | Medical OOP: Consumables | 11 | 8 | 1.01 | 1.83 | 4.54 | 11.23 | 17.66 | 9.40 |
| Passive | Serology: positive; Microscopy: not done/unknown | Medical OOP: Consumables | 9 | 6 | 0.76 | 2.77 | 15.14 | 25.23 | 27.75 | 22.45 |
| Passive | Serology performed; Microscopy negative | Medical OOP: Consumables | 32 | 20 | 0.50 | 0.76 | 1.39 | 11.35 | 63.07 | 10.60 |
| Passive | Confirmed HAT case | Medical OOP: Consumables | 28 | 23 | 0.50 | 1.51 | 3.53 | 11.35 | 104.94 | 9.84 |
| Active | Serology: negative | Medical OOP: Laboratory | 128 | 32 | 0.25 | 0.95 | 2.02 | 4.04 | 20.18 | 3.09 |
| Active | Serology: result unknown | Medical OOP: Laboratory | 10 | 1 | 3.03 | 3.03 | 3.03 | 3.03 | 3.03 | - |
| Active | Serology: positive; Microscopy: not done/unknown | Medical OOP: Laboratory | 13 | 1 | 1.01 | 1.01 | 1.01 | 1.01 | 1.01 | - |
| Active | Serology performed; Microscopy negative | Medical OOP: Laboratory | 61 | 9 | 0.50 | 0.50 | 0.50 | 2.52 | 4.04 | 2.02 |
| Active | Confirmed HAT case | Medical OOP: Laboratory | 18 | 2 | 4.04 | 4.54 | 5.05 | 5.55 | 6.05 | 1.01 |
| Passive | Serology: negative | Medical OOP: Laboratory | 134 | 32 | 0.25 | 1.20 | 1.64 | 2.52 | 28.25 | 1.32 |
| Passive | Serology: result unknown | Medical OOP: Laboratory | 11 | 3 | 0.76 | 1.01 | 1.26 | 2.27 | 3.28 | 1.26 |
| Passive | Serology: positive; Microscopy: not done/unknown | Medical OOP: Laboratory | 9 | 2 | 0.10 | 0.58 | 1.06 | 1.54 | 2.02 | 0.96 |
| Passive | Serology performed; Microscopy negative | Medical OOP: Laboratory | 32 | 8 | 0.76 | 1.01 | 1.51 | 1.77 | 3.53 | 0.76 |
| Passive | Confirmed HAT case | Medical OOP: Laboratory | 28 | 11 | 0.25 | 0.50 | 1.77 | 4.54 | 7.57 | 4.04 |
| Active | Serology: negative | Medical OOP: Other purchases | 128 | 4 | 1.01 | 9.71 | 21.44 | 108.48 | 343.09 | 98.76 |
| Active | Serology: result unknown | Medical OOP: Other purchases | 10 | 0 |  |  |  |  |  |  |
| Active | Serology: positive; Microscopy: not done/unknown | Medical OOP: Other purchases | 13 | 0 |  |  |  |  |  |  |
| Active | Serology performed; Microscopy negative | Medical OOP: Other purchases | 61 | 0 |  |  |  |  |  |  |
| Active | Confirmed HAT case | Medical OOP: Other purchases | 18 | 1 | 6.05 | 6.05 | 6.05 | 6.05 | 6.05 | - |
| Passive | Serology: negative | Medical OOP: Other purchases | 134 | 1 | 75.68 | 75.68 | 75.68 | 75.68 | 75.68 | - |
| Passive | Serology: result unknown | Medical OOP: Other purchases | 11 | 0 |  |  |  |  |  |  |
| Passive | Serology: positive; Microscopy: not done/unknown | Medical OOP: Other purchases | 9 | 0 |  |  |  |  |  |  |
| Passive | Serology performed; Microscopy negative | Medical OOP: Other purchases | 32 | 0 |  |  |  |  |  |  |
| Passive | Confirmed HAT case | Medical OOP: Other purchases | 28 | 3 | 1.01 | 1.26 | 1.51 | 25.98 | 50.45 | 24.72 |
| Active | Serology: negative | Medical OOP: Medication | 128 | 50 | 0.25 | 1.83 | 8.20 | 21.70 | 74.77 | 19.87 |
| Active | Serology: result unknown | Medical OOP: Medication | 10 | 2 | 0.66 | 4.09 | 7.52 | 10.95 | 14.38 | 6.86 |
| Active | Serology: positive; Microscopy: not done/unknown | Medical OOP: Medication | 13 | 2 | 0.25 | 0.95 | 1.64 | 2.33 | 3.03 | 1.39 |
| Active | Serology performed; Microscopy negative | Medical OOP: Medication | 61 | 12 | 0.50 | 2.08 | 5.05 | 7.76 | 27.75 | 5.68 |
| Active | Confirmed HAT case | Medical OOP: Medication | 18 | 2 | 2.02 | 3.91 | 5.80 | 7.69 | 9.59 | 3.78 |
| Passive | Serology: negative | Medical OOP: Medication | 134 | 64 | 0.50 | 6.69 | 12.61 | 17.66 | 85.77 | 10.97 |
| Passive | Serology: result unknown | Medical OOP: Medication | 11 | 7 | 0.50 | 2.77 | 7.57 | 20.31 | 31.79 | 17.53 |
| Passive | Serology: positive; Microscopy: not done/unknown | Medical OOP: Medication | 9 | 4 | 3.43 | 4.64 | 6.56 | 11.73 | 22.70 | 7.09 |
| Passive | Serology performed; Microscopy negative | Medical OOP: Medication | 32 | 12 | 2.02 | 3.28 | 7.32 | 15.51 | 59.54 | 12.24 |
| Passive | Confirmed HAT case | Medical OOP: Medication | 28 | 17 | 1.01 | 2.52 | 8.98 | 46.92 | 121.09 | 44.40 |
| Active | Serology: negative | Medical OOP: Hospitalization | 128 | 10 | 2.52 | 9.71 | 11.60 | 13.37 | 19.17 | 3.66 |
| Active | Serology: result unknown | Medical OOP: Hospitalization | 10 | 0 |  |  |  |  |  |  |
| Active | Serology: positive; Microscopy: not done/unknown | Medical OOP: Hospitalization | 13 | 0 |  |  |  |  |  |  |
| Active | Serology performed; Microscopy negative | Medical OOP: Hospitalization | 61 | 3 | 1.01 | 1.14 | 1.26 | 8.20 | 15.14 | 7.06 |
| Active | Confirmed HAT case | Medical OOP: Hospitalization | 18 | 3 | 6.31 | 6.69 | 7.06 | 8.58 | 10.09 | 1.89 |
| Passive | Serology: negative | Medical OOP: Hospitalization | 134 | 19 | 2.52 | 3.53 | 4.29 | 6.81 | 15.64 | 3.28 |
| Passive | Serology: result unknown | Medical OOP: Hospitalization | 11 | 1 | 3.53 | 3.53 | 3.53 | 3.53 | 3.53 | - |
| Passive | Serology: positive; Microscopy: not done/unknown | Medical OOP: Hospitalization | 9 | 3 | 1.26 | 3.66 | 6.05 | 9.59 | 13.12 | 5.93 |
| Passive | Serology performed; Microscopy negative | Medical OOP: Hospitalization | 32 | 2 | 4.54 | 5.93 | 7.32 | 8.70 | 10.09 | 2.77 |
| Passive | Confirmed HAT case | Medical OOP: Hospitalization | 28 | 9 | 2.52 | 6.05 | 7.57 | 17.66 | 30.27 | 11.60 |
| Active | Serology: negative | Medical OOP: Other | 128 | 10 | 0.10 | 0.28 | 0.50 | 2.40 | 50.45 | 2.12 |
| Active | Serology: result unknown | Medical OOP: Other | 10 | 0 |  |  |  |  |  |  |
| Active | Serology: positive; Microscopy: not done/unknown | Medical OOP: Other | 13 | 1 | 0.35 | 0.35 | 0.35 | 0.35 | 0.35 | - |
| Active | Serology performed; Microscopy negative | Medical OOP: Other | 61 | 1 | 0.50 | 0.50 | 0.50 | 0.50 | 0.50 | - |
| Active | Confirmed HAT case | Medical OOP: Other | 18 | 0 |  |  |  |  |  |  |
| Passive | Serology: negative | Medical OOP: Other | 134 | 30 | 0.50 | 1.51 | 2.77 | 4.41 | 17.66 | 2.90 |
| Passive | Serology: result unknown | Medical OOP: Other | 11 | 2 | 1.01 | 1.39 | 1.77 | 2.14 | 2.52 | 0.76 |
| Passive | Serology: positive; Microscopy: not done/unknown | Medical OOP: Other | 9 | 1 | 25.23 | 25.23 | 25.23 | 25.23 | 25.23 | - |
| Passive | Serology performed; Microscopy negative | Medical OOP: Other | 32 | 3 | 5.05 | 7.32 | 9.59 | 16.15 | 22.70 | 8.83 |
| Passive | Confirmed HAT case | Medical OOP: Other | 28 | 6 | 10.09 | 16.90 | 22.45 | 26.87 | 35.32 | 9.96 |
| Active | Serology: negative | Non-Medical OOP: Transport | 128 | 25 | 0.50 | 2.88 | 5.80 | 9.59 | 37.84 | 6.71 |
| Active | Serology: result unknown | Non-Medical OOP: Transport | 10 | 1 | 1.01 | 1.01 | 1.01 | 1.01 | 1.01 | - |
| Active | Serology: positive; Microscopy: not done/unknown | Non-Medical OOP: Transport | 13 | 0 |  |  |  |  |  |  |
| Active | Serology performed; Microscopy negative | Non-Medical OOP: Transport | 61 | 4 | 0.76 | 0.76 | 1.26 | 1.89 | 2.27 | 1.14 |
| Active | Confirmed HAT case | Non-Medical OOP: Transport | 18 | 5 | 0.20 | 2.52 | 3.03 | 5.05 | 6.56 | 2.52 |
| Passive | Serology: negative | Non-Medical OOP: Transport | 134 | 43 | 0.25 | 1.01 | 1.51 | 5.05 | 17.15 | 4.04 |
| Passive | Serology: result unknown | Non-Medical OOP: Transport | 11 | 5 | 1.01 | 1.51 | 3.03 | 3.53 | 5.05 | 2.02 |
| Passive | Serology: positive; Microscopy: not done/unknown | Non-Medical OOP: Transport | 9 | 1 | 5.05 | 5.05 | 5.05 | 5.05 | 5.05 | - |
| Passive | Serology performed; Microscopy negative | Non-Medical OOP: Transport | 32 | 6 | 0.50 | 2.40 | 3.78 | 4.79 | 7.57 | 2.40 |
| Passive | Confirmed HAT case | Non-Medical OOP: Transport | 28 | 12 | 0.50 | 1.70 | 5.80 | 25.67 | 51.97 | 23.97 |
| Active | Serology: negative | Non-Medical OOP: Food | 128 | 50 | 0.10 | 1.51 | 5.42 | 10.09 | 35.82 | 8.58 |
| Active | Serology: result unknown | Non-Medical OOP: Food | 10 | 1 | 13.62 | 13.62 | 13.62 | 13.62 | 13.62 | - |
| Active | Serology: positive; Microscopy: not done/unknown | Non-Medical OOP: Food | 13 | 0 |  |  |  |  |  |  |
| Active | Serology performed; Microscopy negative | Non-Medical OOP: Food | 61 | 10 | 0.10 | 0.48 | 2.40 | 5.49 | 12.61 | 5.01 |
| Active | Confirmed HAT case | Non-Medical OOP: Food | 18 | 9 | 1.01 | 5.05 | 10.09 | 12.11 | 32.80 | 7.06 |
| Passive | Serology: negative | Non-Medical OOP: Food | 134 | 92 | 0.10 | 1.01 | 4.29 | 10.22 | 70.64 | 9.21 |
| Passive | Serology: result unknown | Non-Medical OOP: Food | 11 | 6 | 4.04 | 5.05 | 7.06 | 11.73 | 16.65 | 6.69 |
| Passive | Serology: positive; Microscopy: not done/unknown | Non-Medical OOP: Food | 9 | 5 | 15.14 | 16.65 | 25.23 | 30.27 | 63.07 | 13.62 |
| Passive | Serology performed; Microscopy negative | Non-Medical OOP: Food | 32 | 15 | 0.50 | 3.78 | 10.60 | 12.61 | 20.18 | 8.83 |
| Passive | Confirmed HAT case | Non-Medical OOP: Food | 28 | 24 | 1.51 | 6.62 | 9.96 | 17.91 | 151.36 | 11.29 |
| Active | Serology: negative | Non-Medical OOP: Accommodation | 128 | 9 | 1.01 | 3.53 | 7.06 | 15.14 | 17.66 | 11.60 |
| Active | Serology: result unknown | Non-Medical OOP: Accommodation | 10 | 0 |  |  |  |  |  |  |
| Active | Serology: positive; Microscopy: not done/unknown | Non-Medical OOP: Accommodation | 13 | 0 |  |  |  |  |  |  |
| Active | Serology performed; Microscopy negative | Non-Medical OOP: Accommodation | 61 | 0 |  |  |  |  |  |  |
| Active | Confirmed HAT case | Non-Medical OOP: Accommodation | 18 | 0 |  |  |  |  |  |  |
| Passive | Serology: negative | Non-Medical OOP: Accommodation | 134 | 4 | 5.30 | 6.62 | 8.83 | 11.73 | 15.14 | 5.11 |
| Passive | Serology: result unknown | Non-Medical OOP: Accommodation | 11 | 1 | 4.29 | 4.29 | 4.29 | 4.29 | 4.29 | - |
| Passive | Serology: positive; Microscopy: not done/unknown | Non-Medical OOP: Accommodation | 9 | 0 |  |  |  |  |  |  |
| Passive | Serology performed; Microscopy negative | Non-Medical OOP: Accommodation | 32 | 0 |  |  |  |  |  |  |
| Passive | Confirmed HAT case | Non-Medical OOP: Accommodation | 28 | 0 |  |  |  |  |  |  |
| Active | Serology: negative | Non-Medical OOP: Other | 128 | 13 | 0.25 | 0.50 | 0.76 | 1.01 | 9.59 | 0.50 |
| Active | Serology: result unknown | Non-Medical OOP: Other | 10 | 0 |  |  |  |  |  |  |
| Active | Serology: positive; Microscopy: not done/unknown | Non-Medical OOP: Other | 13 | 0 |  |  |  |  |  |  |
| Active | Serology performed; Microscopy negative | Non-Medical OOP: Other | 61 | 2 | 0.25 | 0.50 | 0.76 | 1.01 | 1.26 | 0.50 |
| Active | Confirmed HAT case | Non-Medical OOP: Other | 18 | 1 | 0.50 | 0.50 | 0.50 | 0.50 | 0.50 | - |
| Passive | Serology: negative | Non-Medical OOP: Other | 134 | 19 | 0.25 | 0.25 | 0.50 | 1.79 | 151.36 | 1.54 |
| Passive | Serology: result unknown | Non-Medical OOP: Other | 11 | 0 |  |  |  |  |  |  |
| Passive | Serology: positive; Microscopy: not done/unknown | Non-Medical OOP: Other | 9 | 1 | 30.27 | 30.27 | 30.27 | 30.27 | 30.27 | - |
| Passive | Serology performed; Microscopy negative | Non-Medical OOP: Other | 32 | 4 | 3.53 | 6.18 | 9.21 | 17.60 | 36.33 | 11.42 |
| Passive | Confirmed HAT case | Non-Medical OOP: Other | 28 | 5 | 0.50 | 1.01 | 2.52 | 7.57 | 10.09 | 6.56 |
| Active | Serology: negative | Medical OOP | 128 | 57 | 0.25 | 6.05 | 20.18 | 72.40 | 376.14 | 66.35 |
| Active | Serology: result unknown | Medical OOP | 10 | 3 | 0.66 | 2.09 | 3.53 | 17.66 | 31.79 | 15.57 |
| Active | Serology: positive; Microscopy: not done/unknown | Medical OOP | 13 | 3 | 1.26 | 1.89 | 2.52 | 8.00 | 13.47 | 6.10 |
| Active | Serology performed; Microscopy negative | Medical OOP | 61 | 18 | 0.50 | 1.96 | 7.04 | 11.16 | 44.15 | 9.21 |
| Active | Confirmed HAT case | Medical OOP | 18 | 9 | 1.51 | 2.52 | 5.55 | 10.09 | 19.93 | 7.57 |
| Passive | Serology: negative | Medical OOP | 134 | 110 | 0.25 | 6.81 | 17.15 | 26.87 | 242.68 | 20.06 |
| Passive | Serology: result unknown | Medical OOP | 11 | 9 | 1.51 | 5.80 | 18.16 | 28.76 | 34.31 | 22.96 |
| Passive | Serology: positive; Microscopy: not done/unknown | Medical OOP | 9 | 7 | 5.05 | 5.25 | 8.93 | 41.25 | 66.09 | 36.00 |
| Passive | Serology performed; Microscopy negative | Medical OOP | 32 | 20 | 0.76 | 4.29 | 12.87 | 31.60 | 72.65 | 27.31 |
| Passive | Confirmed HAT case | Medical OOP | 28 | 26 | 1.51 | 6.94 | 20.69 | 66.21 | 196.67 | 59.27 |
| Active | Serology: negative | Non-Medical OOP | 128 | 54 | 0.10 | 1.77 | 6.31 | 22.33 | 88.55 | 20.56 |
| Active | Serology: result unknown | Non-Medical OOP | 10 | 1 | 14.63 | 14.63 | 14.63 | 14.63 | 14.63 | - |
| Active | Serology: positive; Microscopy: not done/unknown | Non-Medical OOP | 13 | 0 |  |  |  |  |  |  |
| Active | Serology performed; Microscopy negative | Non-Medical OOP | 61 | 11 | 0.25 | 0.81 | 1.77 | 5.80 | 13.87 | 4.99 |
| Active | Confirmed HAT case | Non-Medical OOP | 18 | 12 | 0.20 | 4.41 | 7.57 | 12.49 | 32.80 | 8.07 |
| Passive | Serology: negative | Non-Medical OOP | 134 | 106 | 0.10 | 1.15 | 5.17 | 12.61 | 160.95 | 11.47 |
| Passive | Serology: result unknown | Non-Medical OOP | 11 | 7 | 1.01 | 6.05 | 9.08 | 12.11 | 24.47 | 6.05 |
| Passive | Serology: positive; Microscopy: not done/unknown | Non-Medical OOP | 9 | 5 | 16.65 | 20.18 | 25.23 | 30.27 | 93.34 | 10.09 |
| Passive | Serology performed; Microscopy negative | Non-Medical OOP | 32 | 17 | 0.50 | 5.55 | 11.10 | 17.66 | 51.21 | 12.11 |
| Passive | Confirmed HAT case | Non-Medical OOP | 28 | 26 | 3.53 | 6.24 | 10.09 | 17.05 | 161.45 | 10.81 |
| Active | Serology: negative | Total OOP | 128 | 63 | 0.10 | 7.64 | 18.52 | 73.97 | 409.18 | 66.32 |
| Active | Serology: result unknown | Total OOP | 10 | 3 | 0.66 | 2.09 | 3.53 | 24.97 | 46.42 | 22.88 |
| Active | Serology: positive; Microscopy: not done/unknown | Total OOP | 13 | 3 | 1.26 | 1.89 | 2.52 | 8.00 | 13.47 | 6.10 |
| Active | Serology performed; Microscopy negative | Total OOP | 61 | 21 | 0.25 | 1.36 | 3.53 | 12.61 | 49.70 | 11.25 |
| Active | Confirmed HAT case | Total OOP | 18 | 14 | 0.20 | 5.17 | 9.84 | 19.49 | 37.59 | 14.32 |
| Passive | Serology: negative | Total OOP | 134 | 119 | 0.25 | 7.44 | 20.18 | 36.45 | 263.37 | 29.01 |
| Passive | Serology: result unknown | Total OOP | 11 | 11 | 1.01 | 6.31 | 18.16 | 31.66 | 58.02 | 25.35 |
| Passive | Serology: positive; Microscopy: not done/unknown | Total OOP | 9 | 7 | 5.45 | 22.12 | 51.46 | 79.59 | 98.39 | 57.47 |
| Passive | Serology performed; Microscopy negative | Total OOP | 32 | 21 | 0.76 | 7.06 | 24.72 | 47.43 | 88.29 | 40.36 |
| Passive | Confirmed HAT case | Total OOP | 28 | 28 | 6.56 | 12.49 | 24.52 | 81.71 | 358.12 | 69.22 |
| Active | Serology: negative | Total OOP excl Food | 128 | 61.00 | 0.25 | 6.05 | 15.14 | 59.54 | 383.96 | 53.48 |
| Active | Serology: result unknown | Total OOP excl Food | 10 | 3.00 | 0.66 | 2.09 | 3.53 | 18.16 | 32.80 | 16.07 |
| Active | Serology: positive; Microscopy: not done/unknown | Total OOP excl Food | 13 | 3.00 | 1.26 | 1.89 | 2.52 | 8.00 | 13.47 | 6.10 |
| Active | Serology performed; Microscopy negative | Total OOP excl Food | 61 | 18.00 | 0.50 | 1.96 | 7.04 | 12.30 | 45.91 | 10.34 |
| Active | Confirmed HAT case | Total OOP excl Food | 18 | 11.00 | 0.20 | 3.53 | 5.05 | 10.85 | 22.96 | 7.32 |
| Passive | Serology: negative | Total OOP excl Food | 134 | 112.00 | 0.76 | 7.00 | 17.53 | 29.45 | 250.76 | 22.45 |
| Passive | Serology: result unknown | Total OOP excl Food | 11 | 11.00 | 1.01 | 3.78 | 12.87 | 24.47 | 41.37 | 20.69 |
| Passive | Serology: positive; Microscopy: not done/unknown | Total OOP excl Food | 9 | 7.00 | 5.05 | 7.19 | 35.32 | 43.77 | 66.09 | 36.58 |
| Passive | Serology performed; Microscopy negative | Total OOP excl Food | 32 | 21.00 | 0.76 | 4.79 | 14.13 | 29.77 | 83.75 | 24.97 |
| Passive | Confirmed HAT case | Total OOP excl Food | 28 | 26.00 | 2.02 | 7.69 | 22.83 | 66.21 | 206.76 | 58.51 |
| Active | Serology: negative | Nb_struct | 128 | 128 | 1.00 | 1.00 | 1.00 | 3.00 | 6.00 | 2.00 |
| Active | Serology: result unknown | Nb_struct | 10 | 10 | 1.00 | 1.00 | 1.50 | 2.00 | 5.00 | 1.00 |
| Active | Serology: positive; Microscopy: not done/unknown | Nb_struct | 13 | 13 | 1.00 | 1.00 | 1.00 | 2.00 | 4.00 | 1.00 |
| Active | Serology performed; Microscopy negative | Nb_struct | 61 | 61 | 1.00 | 1.00 | 1.00 | 2.00 | 4.00 | 1.00 |
| Active | Confirmed HAT case | Nb_struct | 18 | 18 | 1.00 | 1.00 | 2.00 | 2.00 | 5.00 | 1.00 |
| Passive | Serology: negative | Nb_struct | 134 | 134 | 1.00 | 1.00 | 1.00 | 2.00 | 8.00 | 1.00 |
| Passive | Serology: result unknown | Nb_struct | 11 | 11 | 1.00 | 1.00 | 2.00 | 2.00 | 2.00 | 1.00 |
| Passive | Serology: positive; Microscopy: not done/unknown | Nb_struct | 9 | 9 | 1.00 | 1.00 | 1.00 | 3.00 | 3.00 | 2.00 |
| Passive | Serology performed; Microscopy negative | Nb_struct | 32 | 32 | 1.00 | 1.00 | 1.00 | 1.00 | 3.00 | - |
| Passive | Confirmed HAT case | Nb_struct | 28 | 28 | 1.00 | 1.00 | 2.00 | 3.00 | 4.00 | 2.00 |

Supporting Information 17: Frequency of the structures visited by participants

Supporting Information 18: Interview guide – Individual semi-structured interviews

1. **Niveau de connaissance et Perceptions sur la maladie du sommeil :**
a) Connaissez-vous ce qu’on appelle maladie du sommeil ?

Comment appelez-vous cette maladie dans votre langue locale ?

b) Que pensez-vous de la maladie du sommeil dans votre village / milieux de vie ?
c) Pouvez-vous nous parler des endroits où les gens peuvent se faire tester pour la maladie du sommeil ?

Avez-vous déjà vu des équipes mobiles ?

De mini-équipe qui se déplace à Moto ?

Un centre où l’on fait le dépistage de la maladie du sommeil ?

d) Avez-vous déjà participé aux activités de dépistage de la maladie du sommeil ?

(Si non pourquoi ?)

(Si oui, A quelle occasion ?à quel endroit ?)

Que pensez-vous de l’organisation des activités de dépistage de la maladie du sommeil ?

e) Pensez-vous que tous les centres peuvent diagnostiquer la maladie du sommeil ?

Et pourquoi ?

2. **Comportements** des communautés pour la recherche des soins en rapport avec la THA :
   a. Si vous vous sentez malade, que faites-vous d’habitude ?(Demander toujours le pourquoi ?)
   b. A quel moment prendriez-vous la décision d’aller vous faire soigner ?

Attendriez-vous combien de temps avant de chercher à vous faire soigner ? ou à soigner un membre de votre famille ?

c. Où iriez-vous quand vous êtes malades ?

(Orienter vers son itinéraires possibles)

Et si vous pensez à la maladie du sommeil que feriez-vous ?

d. Quand certaines personnes retardent de chercher les soins lorsqu’ils se sentent malades, que peuvent être les raisons ?
e. Que feriez-vous ou recommanderiez-vous à un membre de votre famille si :
   i. Quelqu’un a de la fièvre avec de douleurs articulaires, des prurit ou de maux de tête.
   ii. Quelqu’un présente des troubles du sommeil ou un changement de comportement

3. **Influence du coût sur les choix relatifs au parcours thérapeutique**
   a. Pourriez-vous nous parler des dépenses à engager quand vous devriez rechercher les soins de santé ?(Orienter vers les différents types de dépenses)

- Le coût du déplacement vers le lieu de soins ?
- Le coût de la consultation ?
- Le coût des examens et des médicaments ?
- Les autres coût additionnels(hospitalisation, séjour au centre ,…) ?
  b. Que diriez-vous des dépenses à engager pour les soins en rapport avec la maladie du sommeil ?(Orienter vers les différents types de dépenses)
  c. Est-ce que vous pensez que la participation au dépistage a un impact sur votre travail ?

Lesquels ?

d. Saviez-vous pourquoi certaines personnes retardent de chercher les soins ?
- Pensez-vous que le coût des soins peut constituer une barrière à la cherche des soins de santé ? Et pourquoi ?
- Que peut-on faire pour améliorer cette situation ?
  e. Pensez-vous que le problème d’argent peut être une barrière au dépistage et traitement de la maladie du sommeil ?
- Comment expliquer alors que le dépistage est gratuit ?
- Y-a-t-il d’autres dépenses à faire bien que le dépistage et traitement de la maladie du sommeil sont gratuits ?
- Que peut-on faire pour améliorer cette situation?

4. **Facteurs/coûts importants lors de la prise de décisions concernant le dépistage de la THA.**
   a. Si vous faites des examens dans un centre de santé voisin pour la malaria et pour la maladie du sommeil :

On vous dit que le test de la malaria est négatif mais le test de la maladie du sommeil est positif et qu’il faut aller à l'hôpital général pour faire d’autres examens pour confirmer la maladie du sommeil, **que feriez-vous ? et expliquer pourquoi ?**

b. Si une personne présente les signes de la maladie du sommeil, que doit- elle faire, selon vous ?
c. Rendriez-vous dans un centre de santé ou dans un hôpital général si vous suspectez qu’une personne souffre de la maladie du sommeil ?(Même si vous savez qu'une équipe de dépistage passe parfois dans votre village)
- Pensez-vous que le manque d’argent peut être une barrière ?
- Que peuvent être les autres raisons pour ne pas aller au centre de santé ou à l’hôpital général ?

Supporting Information 19: Interview guide – Focus group discussions

1. **Niveau de connaissance et Perceptions sur la maladie du sommeil :**
a. Connaissez-vous ce qu’on appelle maladie du sommeil ?

Comment appelez-vous cette maladie dans votre langue locale ?

b. Que pensez-vous de la maladie du sommeil dans votre village / milieux de vie ?
c. Pouvez-vous nous parler des endroits où les gens peuvent se faire tester pour la maladie du sommeil ?
d. Pensez-vous que tous les centres peuvent diagnostiquer la maladie du sommeil ?

Et pourquoi ?

2. **Comportements** des communautés pour la recherche des soins en rapport avec la THA :
   a. Si quelqu’un se sent malade, qu’est- ce qu’on fait d’habitude ?(Demander toujours le pourquoi ?Et orienter vers les itinéraires possibles)
   b. Quand certaines personnes retardent de chercher les soins lorsqu’ils se sentent malades, que peuvent être les raisons ?
   c. Que feriez-vous ou recommanderiez-vous à un membre de votre famille si :
      i. Quelqu’un a de la fièvre avec de douleurs articulaires, des de prurit ou de maux de tête.
      ii. Quelqu’un présente des troubles du sommeil ou un changement de comportement
3. **Influence du coût sur les choix relatifs au parcours thérapeutique**
   a. Pourriez-vous nous parler des dépenses à engager quand vous devriez rechercher les soins de santé ?(Orienter vers les différents types de dépenses)

Pensez-vous qu’il y a de différence quand on cherche les soins liés à la maladie du sommeil ? lesquelles ?

b. Est-ce que vous pensez que la participation au dépistage a un impact sur votre travail ?

Lesquels ?

c. Saviez-vous pourquoi certaines personnes retardent de chercher les soins ?
- Pensez-vous que le coût des soins peut constituer une barrière à la cherche des soins de santé ? Et pourquoi ?
- Que peut-on faire pour améliorer cette situation ?
  d. Pensez-vous que le problème d’argent peut être une barrière au dépistage et traitement de la maladie du sommeil ?Comment expliquer ?

Est-ce qu’il existe encore d’autres barrières ?

4. **Facteurs/coûts importants lors de la prise de décisions concernant le dépistage de la THA.**
   a. Si une personne a un test malaria négatif mais un test maladie du sommeil positif, est-ce qu’il irait à l'hôpital général pour d’autres examens de confirmer ? e**t expliquer pourquoi ?**
   b. Rendriez-vous dans un centre de santé ou dans un hôpital général si vous suspectez qu’une personne souffre de la maladie du sommeil ?(Même si vous savez qu'une équipe de dépistage passe parfois dans votre village)

Supporting Information 20: Survey « Evaluation des dépenses médicales et non-médicales THA »

I. **à remplir avant l'entretien**
   I-1 La date de l'entretien

____________________

I-2 Le nom de l'enquêteur

____________________

I-3 **Le nom de la zone de santé**

|  ZS A |  ZS B |  ZS C |
| --- | --- | --- |
|  ZS D |  ZS E |  ZS F |
|  ZS G |  |  |

I-4 Le nom de l'aire de santé

____________________

I-5 Le village ou la ville où l'entretien a eu lieu

____________________

- 5a si autre, veuillez préciser?

____________________

I-6 L'identiﬁant unique de la personne interrogée(Code)

____________________

II. **L'éligibilité**
    II-1 Est-ce que la personne est présente?

*Si la personne n'est pas présente, ﬁxez un RDV à un autre moment et si la personne ne retournera pas à son domicile pendant la période de l'étude, mentionnez-le dans les commentaires à la ﬁn de l'enquête.*

|  Oui |  Non |
| --- | --- |

II-2 Souhaitez-vous participer?

*Si oui, indiquez l'âge et le sexe sur le formulaire de consentement éclairé et laissez la personne signer le formulaire.*

|  Oui |  Non |
| --- | --- |

II-3 L'âge de la personne interrogée

*Seules les personnes âgées de plus de 18 ans sont éligibles pour cette étude. Veuillez lui expliquer que l'entretien ne sera pas poursuivi, si la personne est plus jeune.*

____________________

- II-3a Si non, pourquoi pas

*Une seule réponse possible*

|  Contraintes de temps |  Pas à l'aise |
| --- | --- |
|  Pas intéressé |  Autre |

- II-3b si autre, veuillez préciser

____________________

II-4 Le sexe de la personne interrogée

|  Féminin |  Masculin |
| --- | --- |

II-5 Vous souvenez-vous d'avoir subi un test de dépistage de la maladie du sommeil?

*Seules les personnes qui se souviennent avoir subi un dépistage de la maladie du sommeil sont éligibles pour cette étude. Veuillez luiexpliquer que l'entretien ne sera pas poursuivi, si la personne ne se souvient pas d'avoir été testé.*

|  Oui |  Non |
| --- | --- |

III. **La maladie de sommeil**
     III-1 Que savez-vous de la maladie du sommeil ?

*Laissez la personne énumérer ce qu'elle sait sur la maladie du sommeil sans donner d'indications et indiquez ce qu'elle a mentionné. Plusieurs options sont possibles.*

 La ﬁèvre

 Des maux de tête

 La fatigue

Les douleurs musculaires et articulaires

 La prurite, des démangeaisons

 Les troubles d'appétit et/ou l'amaigrissement

 Des troubles du sommeil

 Des troubles de comportement

Des troubles neurologiques

La perte de conscience, le coma

Une maladie mortelle

 Mouche tsetse

 Autre

 Rien

- 1b si autre, veuillez préciser?

____________________

III-2 Connaissez-vous quelqu'un qui a eu la maladie du sommeil?

|  Oui |  Non |
| --- | --- |

- 2a Si oui, comment connaissez-vous cette personne/ces personnes ?

*Plusieurs options sont possibles*

 Un membre de la famille

 Un voisin

 Quelqu'un du même village/de la même ville

Autre

- 2b si autre, veuillez préciser?

____________________

III-3 Où peut-on se faire tester pour la maladie du sommeil ?

*Laissez la personne énumérer tous les endroits où, selon elle, vous pouvez vous faire tester pour la maladie du sommeil, sans donner d'indications et indiquez ce qu'elle a mentionné. Plusieurs options sont possibles*

Tradipraticiens

Pharmacie

Poste de santé - Centre de santé - Centre de référence

Hôpital sécondaire - Hôpital Général de Référence

 Centre privé - cabinet médical privé

Une équipe mobile avec un véhicule

 Une équipe mobile avec des motos

 Centre de dépistage, diagnostic, traitement et controle(CDTC)

 Autre

- 3a si autre, veuillez préciser?

____________________

IV. **Le parcours de recherche de soins**
    IV-1 Etiez-vous soufrant au moment du dépistage?

|  Oui |  Non |
| --- | --- |

- 1a Si oui, quel type de symptômes avez-vous eu?

*Laissez la personne énumérer tous les symptômes sans donner d'indications et indiquez ce qu'elle a mentionné. Plusieurs options sont possibles*

 La ﬁèvre

 Des maux de tête

 La fatigue

Les douleurs musculaires et articulaires

 La prurite, des démangeaisons

 Les troubles d'appétit et/ou l'amaigrissement

 Des troubles du sommeil

 Des troubles de comportement

Des troubles neurologiques

 Autre

- IV-1b si autre, veuillez préciser?

____________________

IV-2 Avais-vous déjà consulté d'autres formations sanitaires pour ces symptomes avant d'être dépisté pour la maladie du sommeil

|  Oui |  Non |
| --- | --- |

- IV-2a Si oui, lesquels?

*Plusieurs options sont possibles*

Tradipraticiens

Pharmacie

Poste de santé - Centre de santé - Centre de référence

Hôpital sécondaire - Hôpital Général de Référence

 Centre privé - cabinet médical privé

Une équipe mobile avec un véhicule

 Une équipe mobile avec des motos

 Centre de dépistage, diagnostic, traitement et controle(CDTC)

 Autre

- IV-2b si autre, veuillez préciser

____________________

- IV-2c et dans quel ordre?
  *Classement en appuyant longuement sur un choix et en le faisant glisser pour changer de position. Classez uniquement les choix énumérés dans la question précédente. Les autres choix peuvent être laissés en bas*

Tradipraticiens

Pharmacie

Poste de santé - Centre de santé - Centre de référence

Hôpital sécondaire - Hôpital Général de Référence

Centre privé - cabinet médical privé

Une équipe mobile avec un véhicule

Une équipe mobile avec des motos

Centre de dépistage, diagnostic, traitement et controle(CDTC)

Autre

IV-3 Participez-vous à un système d'assurance maladie ?

|  Oui |  Non |
| --- | --- |

- IV-3a Si oui, comment cela est-il ﬁnancé ?

|  Travail |  Initiative locale |
| --- | --- |
|  Autre |  |

- IV-3b si autre, veuillez préciser

____________________

V. **Le dépistage sérologique**
   V-1 Vous souvenez-vous de la date approximative à laquelle le premier test du dépistage a eu lieu?

*Indiquez le 1er du mois si la personne ne se souvient que du mois où le test a eu lieu.*

______ yyyy-mm-dd______________

V-2 Où avez-vous subi ce test?

*Une seule réponse possible*

Tradipraticiens

Pharmacie

Poste de santé - Centre de santé - Centre de référence

Hôpital sécondaire - Hôpital Général de Référence

 Centre privé - cabinet médical privé

Une équipe mobile avec un véhicule

 Une équipe mobile avec des motos

 Centre de dépistage, diagnostic, traitement et controle(CDTC)

 Autre

- IV-2a si autre, veuillez préciser

____________________

- IV-2b Connaissez-vous le nom de l'établissement ou de l'équipe qui a eﬀectué le test ?

____________________

V-3 Avez-vous été informé des résultats du premier test?

|  Oui |  Non |
| --- | --- |

V-4 Quel était le résultat?

|  Négatif |  Positif |
| --- | --- |

V-5 Avez-vous oﬀert quelque chose à l'équipe mobile lorsqu'elle a terminé le dépistage dans votre village?

|  Oui |  Non |
| --- | --- |

- IV-5a si oui, quoi

____________________

VI. **Les tests de microscopie**
    VI-1 **Ont-ils recommandé des tests supplémentaires ?**

|  Oui |  Non |
| --- | --- |

- VI-1a Auriez-vous fait des tests supplémentaires s'ils avaient été recommandés?

| Oui | Non |
| --- | --- |

- VI-1b si non pourquoi pas

____________________

- VI-1c Même si vous aviez dû vous déplacer vers un autre structure de santé ?

| Oui | Non |
| --- | --- |

- VI-1d si non pourquoi pas

____________________

VI-2 **Si oui, Ont-ils expliqué pourquoi vous aviez besoin de tests supplémentaires?**

| Oui | Non |
| --- | --- |

VI-3 **Quelle était la raison des tests supplémentaires?**

____________________

VI-4 **Où deviez-vous vous rendre pour les tests supplémentaires?**

*Une seule réponse possible*

Une personne vous rendra visite plus tard à votre domicile pour eﬀectuer des tests de suivi.

Le test de conﬁrmation s'est déroulé au même endroit que le premier test.

Vous avez dû vous rendre dans un autre centre de santé/hôpital.

Autre

- VI-4a si autre, veuillez préciser

____________________

VI-5 **Avez-vous fait des tests supplémentaires?**

| Oui | Non |
| --- | --- |

- VI-5a Si non, pourquoi pas?

*Plusieurs options sont possibles*

| Contraintes de temps | Pas à l'aise |
| --- | --- |
| Pas intéressé | Autre |

- VI-5b si autre, veuillez préciser

____________________

VI-6 **Avez-vous été informé des résultats des tests supplémentaires?**

| Oui | Non |
| --- | --- |

VI-7 **Quel était le résultat?**

| Négatif | Positif |
| --- | --- |

VII. **Le traitement**
     VII-1 Ont-ils recommandé de suivre un traitement contre la maladie du sommeil?

| Oui | Non |
| --- | --- |

- VII-1a Auriez-vous suivi un traitement si cela avait été recommandé ?

| Oui | Non |
| --- | --- |

- VII-1b si non pourquoi pas?

____________________

VII-2 Si oui, Ont-ils expliqué pourquoi vous aviez besoin d'un traitement?

| Oui | Non |
| --- | --- |

VII-3 Quelle était la raison?

____________________

VII-4 Où deviez-vous vous rendre pour le traitement?

*Une seule réponse possible*

Tradipraticiens

Pharmacie

Poste de santé - Centre de santé - Centre de référence

Hôpital sécondaire - Hôpital Général de Référence

Centre privé - cabinet médical privé

Une équipe mobile avec un véhicule

Une équipe mobile avec des motos

Centre de dépistage, diagnostic, traitement et controle(CDTC)

Autre

- VII-4a si autre, veuillez préciser

____________________

VII-5 Avez-vous cherché un traitement?

| Oui | Non |
| --- | --- |

- VII-5a Pourquoi n'êtes-vous pas allé vous faire soigner ?

*Notez la raison*

____________________

VII-6 Où êtes-vous allé pour le traitement?

___________________

VII-7 Est-ce le même endroit que celui qui a été recommandé lors des tests de microscopie?

| Oui | Non |
| --- | --- |

- VII-7a Pourquoi vous y êtes-vous rendu si ce lieu est diﬀérent de ce qui avait été recommandé lors des tests de microscopie?

*Plusieurs options sont possibles*

Un accès plus facile

Moins cher

Mieux connu, plus de conﬁance

Famille dans le même village

Autre

- VII-7b si autre, veuillez préciser

____________________

VII-8 Quelqu'un vous a-t-il accompagné pendant le traitement?

| Oui | Non |
| --- | --- |

VII-9 Si oui, quelle était la relation de la personne qui vous a accompagné?

Parent

Enfant

Autre membre de la famille

Autre

VII-10 Connaissez-vous le nom du traitement que vous avez reçu?

*Une seule réponse possible*

| Oui | Non |
| --- | --- |

- VII-10a Si oui, quel était le nom du traitement ?

*Une seule réponse possible*

Pentamidine

NECT

Fexinidazole

Acoziborole

Autre

- VII-10b si autre, veuillez préciser

____________________

VII-11 Avez-vous dû attendre pour commencer votre traitement lorsque vous êtes arrivé au centre de traitement ? Si oui, combien de jours ?

*Mettre 0 si la personne n'a pas eu à attendre*

____________________

VII-12 Combien de jours a duré le traitement?

*Sans compter le temps d'attente*

____________________

VII-13 Etiez vous hospitalisé pendant le traitement?

| Oui | Non |
| --- | --- |

VII-14 Avez-vous dû y retourner une fois le traitement terminé pour des RDV de suivi?

| Oui | Non |
| --- | --- |

VII-15 Est-ce que quelqu'un vous a rendu visite à domicile pour une visite de suivi?

| Oui | Non |
| --- | --- |

VIII. **Les Déplacements**
      VIII-1 Avez-vous dû vous déplacer pour vous faire le premier test de dépistage?

| Oui | Non |
| --- | --- |

- 1a Combien de temps avez-vous dû voyager?

*Une seule réponse possible*

Moins de 30 minutes

Moins d'une heure

Moins de 2 heures

Moins de 4 heures

Moins de 8 heures

Plus d'un jour, j'ai dû passer la nuit quelque part en chemin.

- VIII-1b Comment avez-vous dû voyager?

*Plusieurs options sont possibles*

A pied

En vélo

Par moto

En voiture

Autre

- VIII-1c si autre, veuillez préciser

____________________

- VIII-1d Si vous avez dû utiliser un moyen de transport, avez-vous utilisé votre propre moyen de transport ?

| Oui | Non |
| --- | --- |

VIII-2 Les tests de microscopie ont-ils été eﬀectués immédiatement sur place après le premier test ?

| Oui | Non |
| --- | --- |

VIII-3 Si Non, avez-vous dû vous déplacer pour les tests de suivi ?

| Oui | Non |
| --- | --- |

- VIII-3a Combien de temps avez-vous dû voyager?

*Une seule réponse possible*

Moins de 30 minutes

Moins d'une heure

Moins de 2 heures

Moins de 4 heures

Moins de 8 heures

Plus d'un jour, j'ai dû passer la nuit quelque part en chemin.

- VIII-3b Comment avez-vous dû voyager?

*Plusieurs options sont possibles*

A pied

En vélo

Par moto

En voiture

Autre

- VIII-3c si autre, veuillez préciser

____________________

- VIII-3d Si vous avez dû utiliser un moyen de transport, avez-vous utilisé votre propre moyen de transport ?

| Oui | Non |
| --- | --- |

VIII-4 Avez-vous dû vous déplacer pour le traitement en plus des déplacements liés au test de micropscopie?

| Oui | Non |
| --- | --- |

- VIII-4a Combien de temps avez-vous dû voyager?

*Une seule réponse possible*

Moins de 30 minutes

Moins d'une heure

Moins de 2 heures

Moins de 4 heures

Moins de 8 heures

Plus d'un jour, j'ai dû passer la nuit quelque part en chemin.

- VIII-4b Comment avez-vous dû voyager?

*Plusieurs options sont possibles*

A pied

En vélo

Par moto

En voiture

Autre

- VII-4c si autre, veuillez préciser

____________________

- VIII-4d Si vous avez dû utiliser un moyen de transport, avez-vous utilisé votre propre moyen de transport ?

Votre propre moyen de transport

Une équipe THA

Particulier

- VII-4e si autre, veuillez préciser

____________________

IX. **Les dépenses**
    IX-1 Avez-vous dépensé de l'argent lors du dépistage de la maladie du sommeil ou lors des visites précédentes ?

*Complétez le tableau*

| Oui | Non |
| --- | --- |

IX-2 Environ combien d'argent et de temps avez-vous dépensé pour chaque visite(Avant & pendant la détection et la gestion des cas)? Complétez le tableau et indiquez à nouveau le numéro mentionné dans le registre ci-dessous.

____________________

IX-3 Avez-vous perdu un revenu lors du dépistage de la THA ?

| Oui | Non |
| --- | --- |

IX-4 Avez-vous perdu un revenu lors des tests microscopiques supplémentaires ?

| Oui | Non |
| --- | --- |

IX-5 Avez-vous perdu un revenu lors du traitement ?

| Oui | Non |
| --- | --- |

IX-6 Quelle est/sont votre/vos activité(s) génératrice(s) de revenu principaux?

*Plusieurs options sont possibles*

L'élevage d'animaux domestiques - la vente de bétail

La pêche - la vente de poissons

Agriculture - la vente des recoltes

La vente de l'huile de palme

La vente du vin de palme

La main-d'œuvre occasionnelle - le secteur agricole

La main-d'œuvre occasionnelle - le secteur non agricole

La vente des marchandises, le petit commerce et l'artisanat

La vente des marchandises, le petit commerce et l'artisanat

Propre entreprise

Un salarié du gouvernement

Aucune source de revenu

Autre

IX-6a si autre, veuillez préciser

____________________

IX-7 Une personne de votre foyer vous a-t-elle accompagné lors des tests sérologiques

| Oui | Non |
| --- | --- |

- IX-7b Une personne de votre foyer vous a-t-elle accompagné lors des tests microscopiques supplémentaires

| Oui | Non |
| --- | --- |

- IX-7cUne personne de votre foyer vous a-t-elle accompagné lors du traitement

| Oui | Non |
| --- | --- |

- IX-7d Si oui, cette personne a-t-elle perdu un revenu pendant cette période ?

| Oui | Non |
| --- | --- |

- IX-7e Quelle est l'activité principale de la personne qui vous a accompagné? *Plusieurs options sont possibles*

L'élevage d'animaux domestiques - la vente de bétail

La pêche - la vente de poissons

Agriculture - la vente des recoltes

La vente de l'huile de palme

La vente du vin de palme

La main-d'œuvre occasionnelle - le secteur agricole

La main-d'œuvre occasionnelle - le secteur non agricole

La vente des marchandises, le petit commerce et l'artisanat

La vente des marchandises, le petit commerce et l'artisanat

Propre entreprise

Un salarié du gouvernement

Aucune source de revenu

Autre

- IX-7f si autre, veuillez préciser

____________________

IX-8 Comment avez-vous ﬁnancé les coûts liés au dépistage et/ou au traitement

*Plusieurs options sont possibles*

épargne

emprunt d'argent

vente de biens

contributions de la famille et des amis

autres

- IX-8a si autre, veuillez préciser

____________________

IX-9 Combien cotisez-vous à l'assurance maladie(FC)?

____________________

IX-10 Cet à quelle fréquence contribuez-vous ?

mensuellement

tous les 2 mois

tous les 3 mois

tous les 6 mois

annuellement

Autre

- IX-10a si autre, veuillez préciser

____________________

X. **L'impact sur le comportement**
   X-1 Ces coûts ont-ils inﬂuencé votre comportement à l'égard du dépistage et/ou du traitement de la THA

| Oui | Non |
| --- | --- |

X-2 Si oui, comment ?

____________________

X-3 Recommanderiez-vous à quelqu'un de se faire tester pour la maladie du sommeil

| Oui | Non |
| --- | --- |

X-4 Pourquoi pas

____________________

X-5 Où leur conseilleriez-vous d'aller

*Plusieurs options sont possibles*

Tradipraticiens

Pharmacie

Poste de santé - Centre de santé - Centre de référence

Hôpital sécondaire - Hôpital Général de Référence

Centre privé - cabinet médical privé

Une équipe mobile avec un véhicule

Une équipe mobile avec des motos

Centre de dépistage, diagnostic, traitement et controle(CDTC)

Autre

- X-5a si autre, veuillez préciser

____________________

Merci pour votre coopération ! Avez-vous encore des questions ou des commentaires?

II-1 Avez-vous dépensé de l'argent lors du dépistage de la maladie du sommeil ou lors des visites précédentes ? => Tableau

Supporting Information 21: GRAMMS compliance table

| **GRAMMS criterion** | **Where addressed in manuscript** | **Pg** |
| --- | --- | --- |
| 1. Justification for mixed methods | Study design | 5 |
| 2. Design: purpose, priority and sequence | Methods: Study design | 5 |
| 3. Sampling, data collection and analysis for each method | Methods:   - Qualitative phase; - Quantitative phase | 5-7 |
| 4. Where and how integration occurred | Integration of qualitative and quantitative findings | 5 |
| 5. Limitations of combining methods | Discussion: Strengths and limitations | 14-16 |
| 6. Insights gained from integration | Results/Discussion: interpretation of care-seeking pathways, financial barriers and passive screening costs | 14-16 |

**References**

1. World Health Organization (WHO). Human African trypanosomiasis: Number of new reported cases of Human African trypanosomiasis (T.b. gambiense):2024 Geneva: WHO; 2024 [updated 01/07/2025; cited 2025 08/08/2025]. Available from: <https://apps.who.int/neglected_diseases/ntddata/hat/hat.html>
2. Snijders R. Study Area: Zenodo; 2024. doi: <https://doi.org/10.5281/zenodo.12624925>.

1. Motorcycle of the health district or the HAT screening mobile team
2. Note: Type of health structure was a highly significant predictor of cost level (p < 0.001). Individual coefficient p-values are not significant due to high variance in OOP costs and small sample sizes within mobile team reference category. Exp(β) represents the multiplicative effect on costs relative to mobile team screening.
