# Supplementary material for: The household burden of sleeping sickness: out-of-pocket costs for diagnosis and treatment
Source: BMJ Glob Health. 2026 Oct 16;11(10):e025040. doi: 10.1136/bmjgh-2026-025040 (PMC13691789; doi:10.1136/bmjgh-2026-025040)
Supplement: Supplementary file 2 [file bmjgh-11-10-s002.docx]

### BMJ Global Health Author Reflexivity Statement

Adapted from Morton, B., Vercueil, A., Masekela, R., Heinz, E., Reimer, L., Saleh, S., Kalinga, C., Seekles, M., Biccard, B., Chakaya, J., Abimbola, S., Obasi, A. and Oriyo, N. (2022), Consensus statement on measures to promote equitable authorship in the publication of research from international partnerships. Anaesthesia, 77: 264-276. <https://doi.org/10.1111/anae.15597>

| **Study conceptualisation** | |
| --- | --- |
| 1. How does this study address local research and policy priorities? | The elimination of gambiense Human African Trypanosomiasis remains a priority disease for the DRC national control programme (PNLTHA) and WHO’s 2030 NTD Roadmap. Understanding the financial barriers faced by patients and communities is directly relevant to national effective and equitable elimination strategies and informs health system planning for the transition from active to passive screening. The study was designed in response to an identified gap in evidence needed to guide programme decisions in high-burden, low-resource settings. |
| 2. How were local researchers involved in study design? | Researchers from the PNLTHA (DRC national sleeping sickness control programme) were co-investigators and were involved from the outset in study conceptualisation, development of the study protocol, selection of study sites, development of data collection tools, implementation of the study and interpretation of results. |
| **Research management** | |
| 3. How has funding been used to support the local research team(s)? | Funding supported implementation of the field study in DRC, including local field logistics, engagement of survey teams and supervisors, qualitative interviews and focus group discussions, quantitative data collection across six health zones, and collaboration with PNLTHA. The manuscript states that DGD funded work within a project aiming to eliminate HAT in DRC, while Gates Foundation funding supported the HAT MEPP modelling/economic work.  Funding supported the salaries of local surveyors and field costs in the DRC-based research staff. Travel, accommodation, and operational costs for field teams, supervisors and PNLTHA researchers were covered throughout the study period. |
| **Data acquisition and analysis** | |
| 4. How are research staff who conducted data collection acknowledged? | All Congolese and non-Congolese contributors who met authorship criteria are included as co-authors; other contributors, including local surveyors, field supervisors, and the experienced Kikongo-speaking anthropologist who conducted qualitative interviews and focus group discussions are acknowledged by name in the acknowledgements section of the manuscript. Their essential contribution to data collection in remote communities is explicitly recognised. |
| 5. How have members of the research partnership been provided with access to study data? | Data access was managed in line with ethical and privacy requirements. Study data are retained at the Institute of Tropical Medicine, Antwerp, Belgium and are not openly accessible because of ethical privacy concerns. All DRC-based and non-DRC-based co-investigators (PNLTHA) have access to the study dataset. Within the partnership, data and summary analyses were shared as needed for analysis, interpretation and manuscript development. |
| 6. How were data used to develop analytical skills within the partnership? | DRC-based co-investigators participated in protocol development, field training, data analysis, interpretation of findings and manuscript development. These activities linked field experience from PNLTHA with health-economic analysis expertise from ITM and Swiss TPH. |
| **Data interpretation** | |
| 7. How have research partners collaborated in interpreting study data? | The study was conducted through collaboration between DRC programme partners and international academic partners. Local partners contributed contextual understanding of the HAT care pathway, health-zone organisation, active and passive screening practices, patient referral routes, and practical barriers faced by communities. This contextual interpretation guided the selection of study sites, data collection and interpretation of study results. It was important for the interpretation of care-seeking behaviour, OOP expenditures, and barriers to screening participation. |
| **Drafting and revising for intellectual content** | |
| 8. How were research partners supported to develop writing skills? | The primary author (RS) drafted the first version of the manuscript afterwards all DRC-based and non-DRC-based co-investigators were invited to review manuscript drafts, contribute to interpretation of findings, refine programme and policy implications, and approve the final content. Senior academic partners from the DRC and Swiss TPH provided mentorship on protocol development, analysis, scientific writing and contextual and health-economic interpretation. |
| 9. How will research products be shared to address local needs? | Findings will be shared with the PNLTHA and relevant DRC and international HAT stakeholders to inform programme planning, particularly around the financial burden of HAT screening and treatment can affect effectiveness and affordability of HAT screening and elimination strategies. |
| **Authorship** | |
| 10. How is the leadership, contribution and ownership of this work by LMIC researchers recognised within the authorship? | Four of the eight authors are DRC-based researchers from the PNLTHA (AF, PN, EM, AM), reflecting the substantial contribution of local partners to study design, data collection, and interpretation. Their authorship positions reflect their intellectual contribution to the work. One senior PNLTHA authors (AM) shares equal senior authorship alongside the European partners. |
| 11. How have early career researchers across the partnership been included within the authorship team? | The first author (RS) is an early career researcher who led the study design, data collection, analysis, and manuscript preparation as part of a PhD trajectory. Early career DRC-based researchers (AF, PN) are included as co-authors in recognition of their contributions to data collection and local contextualisation of findings. |
| 12. How has gender balance been addressed within the authorship? | The authorship team includes both male and female researchers. The lead author is female, and one of the last authors. |
| **Training** | |
| 13. How has the project contributed to training of LMIC researchers? | Local field surveyors and supervisors received structured training in data collection, survey administration using ODK. This training enhanced individual capacity within the PNLTHA team and contributed to strengthening research skills within the DRC ministry of health. |
| **Infrastructure** | |
| 14. How has the project contributed to improvements in local infrastructure? | The study did not contribute to the strengthening of local physical infrastructure but contributed to strengthening the evidence infrastructure for HAT control in the DRC |
| **Governance** | |
| 15. What safeguarding procedures were used to protect local study participants and researchers? | The study received ethical approval from the Institutional Review Board of the Institute of Tropical Medicine, Antwerp (ref. 1493/21) and the Ethics Committee of the Université Protestante au Congo, DRC (ref. CEUPC 0091). All participants provided written informed consent before enrolment. Interviews and focus groups were conducted by trained staff in the local language, and data access is restricted because of privacy concerns. De-identified data can only be accessed through a motivated request and under conditions preventing publication of transcripts or survey results in whole or in part. |
